# Nur77-IRF1 axis inhibits esophageal squamous cell carcinoma growth and improves anti-PD-1 treatment efficacy

**DOI:** 10.1038/s41420-024-02019-x

**Published:** 2024-05-24

**Authors:** Huanying Shi, Lu Chen, Tianxiao Wang, Wenxin Zhang, Jiafeng Liu, Yuxin Huang, Jiyifan Li, Huijie Qi, Zimei Wu, Yi Wang, Haifei Chen, Yongjun Zhu, Qunyi Li

**Affiliations:** 1 Department of Pharmacy, Huashan Hospital, Fudan University, No.12 Urumqi Middle Road, Shanghai, 200040 China; 2 Department of Cardio-Thoracic Surgery, Huashan Hospital, Fudan University, No.12 Urumqi Middle Road, Shanghai, 200040 China

**Keywords:** Oesophageal cancer, Tumour biomarkers, Cell death and immune response

## Abstract

The nuclear receptor Nur77 plays paradoxical roles in numerous cancers. However, whether Nur77 inhibits esophageal squamous cell carcinoma (ESCC) growth and affects immunological responses against ESCC has not been determined. The functional role of Nur77 in ESCC was investigated in this study using human ESCC cell lines, quantitative real-time polymerase chain reaction (PCR), cell proliferation and colony formation assays, flow cytometry analysis, western blotting and animal models. The target gene controlled by Nur77 was verified using dual-luciferase reporter assays, chromatin immunoprecipitation analysis and functional rescue experiments. To examine the clinical importance of Nur77, 72 human primary ESCC tissues were subjected to immunohistochemistry. Taken together, these findings showed that, both in vitro and in vivo, Nur77 dramatically reduced ESCC cell growth and triggered apoptosis. Nur77 directly interacts with the interferon regulatory factor 1 (IRF1) promoter to inhibit its activity in ESCC. Pharmacological induction of Nur77 using cytosporone B (CsnB) inhibited ESCC cell proliferation and promoted apoptosis both in vitro and in vivo. Furthermore, CsnB increased CD8+ T-cell infiltration and cytotoxicity to inhibit the formation of ESCC tumors in an immunocompetent mouse model. In ESCC tissues, Nur77 expression was downregulated, and IRF1 expression was increased; moreover, their expression levels were negatively related. IRF1 and Nur77 were strongly correlated with overall survival. These findings suggested that Nur77 targets and regulates the IRF1/PD-L1 axis to serve as a tumor suppressor in ESCC.

**Figure:**
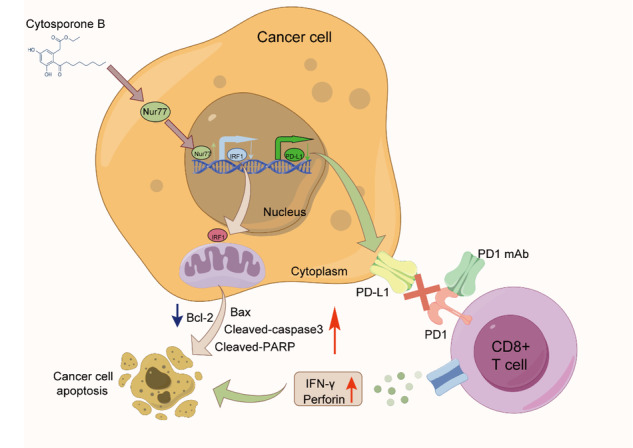
Graphical abstract of the regulatory mechanism of Nur77 overexpression downregulates IRF1 in the inhibition of ESCC progression and enhance anti-PD-1 therapy efficacy.

Graphical abstract of the regulatory mechanism of Nur77 overexpression downregulates IRF1 in the inhibition of ESCC progression and enhance anti-PD-1 therapy efficacy.

## Introduction

Esophageal cancer (EC) is a very aggressive malignant tumor that ranks eighth in terms of cancer incidence and sixth in terms of cancer-related death [1]. Based on its pathological features, esophageal squamous cell carcinoma (ESCC) and esophageal adenocarcinoma (EAC) are the two main histological subtypes of EC [2]. The most common type, ESCC, makes up 90% of cases of EC worldwide and is more common in East Asian and Middle Eastern nations, particularly in China, where it has the highest morbidity and mortality rate. Since early ESCC symptoms are inconspicuous, almost 70% of patients are diagnosed at an intermediate or advanced stage, when their 5-year survival chances range from 5 to 20%. In contrast, early ESCC survival rates are generally good [3]. The conventional treatment for patients with locally advanced ESCC is neoadjuvant or definitive chemoradiotherapy (CRT); however, the clinical outcomes are still unsatisfactory, and only a small percentage of patients benefit from this therapy [4]. In addition, medication targeting VEGF, HER-2, or EGFR was not able to significantly reduce ESCC incidence [5], in part because intratumoral heterogeneity or specific biomarkers were not present. The search for novel ESCC therapeutic approaches, particularly immunotherapeutic-targeted medicines, was prompted by the poor improvement in treatment outcomes associated traditional medications.

Immunotherapy with immune checkpoint inhibitors (ICIs), such as cytotoxic T-lymphocyte-associated protein-4 (CTLA4), programmed death-1 (PD-1), and programmed death-ligand 1 (PD-L1), has improved the clinical outcomes of ESCC patients significantly in recent years [6]. According to previous studies, compared with chemotherapy, PD-1 inhibitor monotherapy has demonstrated greater antitumor efficacy as a second-line therapy for advanced ESCC with controllable safety [7]. However, only a limited number of ESCC patients benefit from monoimmunotherapy, and the mechanisms underlying immunotherapy resistance are generally unclear [8]. For advanced ESCC, ICIs combined with chemotherapy are currently recommended as first-line treatments rather than chemotherapy alone [7, 9, 10]. The combination of ICIs and CRT has emerged as a novel approach with potential for cooperative action and increased efficacy in locally advanced ESCC patients. The prognosis for patients with ESCC is still poor even with the development of interdisciplinary treatments such as immunotherapy, radiation, chemotherapy, and surgery. To identify patients who are likely to respond to these medications and to identify combination therapies that will overcome drug resistance and increase survival in ESCC patients, innovative biomarkers should be identified as soon as possible.

Nur77 (also called NR4A1, TR3, or NGFI-B) is an orphan nuclear receptor encoded by the immediate early gene Nr4a1 that belongs to the steroid/thyroid/retinoid superfamily [11] and is involved in cell proliferation, differentiation, and apoptosis [12]. Nur77 is a transcription factor that regulates gene expression by binding to its target gene promoter. Moreover, it plays a paradoxical role in the genesis of several malignancies. Numerous studies have shown that Nur77 can either promote or suppress cancer formation, depending on the cell type, cellular milieu, and subcellular location [12]. Nur77 shields TPβ in the mitochondria from oxidation, which promotes melanoma cell growth under metabolic stress [13]. The expression of Nur77 is positively correlated with advanced-stage colorectal cancer, distant metastasis, and poor patient prognosis [14]. In human pancreatic cancer, Nur77 is overexpressed, and pancreatic cancer cell and tumor development are inhibited by Nur77 inactivation [15]. In bladder cancer cells, downregulating Nur77 via the use of methylene-substituted diindolylmethanes triggers apoptosis and inhibits tumor growth [16]. By blocking mTORC1 signaling, the deletion of Nur77 slowed tumor development and caused death in lung cancer cells and lung tumors in murine orthotopic and metastatic models [17]. Nur77 was highly expressed in a subset of high grade serous ovarian cancer samples from patients with worse progression-free survival [18]. The characteristics of cancer stem cells are favorably linked to increased Nur77 expression in gastric cancer cells [19]. Nonetheless, Nur77 also has a tumor-suppressive role. Nur77 causes HCC cells to produce the lncRNA WFDC21P, which suppresses HCC cell growth and metastasis in vitro and in vivo [20]. Nur77 could significantly suppress the growth of intestinal epithelial cells through inhibiting Wnt signaling activity [21]. Acute myeloid leukemia develops in mice when Nur77 is disrupted [22]. However, Nur77 performs unique or even contradictory regulatory roles in breast cancer [23–27]. Although abnormal expression of Nur77 is common in many human malignancies, its exact role in the formation of ESCC has yet to be determined; hence, further investigation into the role and mechanism of Nur77 in ESCC is necessary.

Notably, Nur77 is also essential for the mechanisms involved in antitumor immunity [28, 29]. As previously mentioned, Nur77 knockout enhances the antitumor effect of CAR-T cells on a number of tumors. Examination of lymphocytes that had infiltrated the tumor revealed that Nur77 was overexpressed in fatigued T cells, and that Nur77 was associated with increased expression of Tim3 and PD-L1, decreased expression of cytokines, and decreased levels of cell death [30]. As an essential mediator of T-cell failure, Nur77 deletion partially reverses exhaustion, leading to tumor regression and enhanced survival [31]. Additionally, through the IFN-γ/p-STAT1/IRF1 pathway, Nur77 causes NK-cell dysfunction in hepatocellular carcinoma [32]. High expression of Nur77 prevents invariant NK T cell growth and function [33]. Inhibition of Nur77 enhances anticancer immunity through disruption of Treg-mediated immunological tolerance [34]. This effect was also observed in both xenograft and syngeneic mouse models in which a Nur77 antagonist was used to induce PD-L1 degradation and decrease the formation of mammary tumors [35]. In addition, a recent study revealed that Nur77 is a predictive biomarker that corresponds with immune infiltration in patients with prostate adenocarcinoma [36]. However, whether Nur77 alters immunity to influence the progression and outcome of ESCC requires further exploration.

In the present study, we discovered that Nur77 is a tumor suppressor that causes ESCC cells to undergo apoptosis and inhibit proliferation. We demonstrated that Nur77 suppresses ESCC cell lines and animal xenograft models both in vitro and in vivo. Nur77 inhibited IRF1 expression. The small-molecule substance cytosporone B (CsnB) is a potent and selective Nur77 agonist [37]. Treatment with CsnB caused apoptosis and successfully stopped ESCC cell growth. Furthermore, we found that pharmacological stimulation of Nur77 significantly enhances anti-PD-1 immune treatment efficacy in a mouse tumor model. Clinically, Nur77 expression was downregulated in ESCC tissues whereas IRF1 expression was increased, and their expression was negatively related. Nur77 and IRF1 were also found to be strongly related to overall survival (OS). Taken together, these findings might provide additional support for the hypothesis that Nur77 could serve as a predictive biomarker for ESCC patients and that Nur77 might be controlled as a treatment target for this disease.

## Results

### Nur77 inhibited ESCC cell proliferation in vitro and ESCC cell tumorigenesis in vivo

**Fig. 1:**
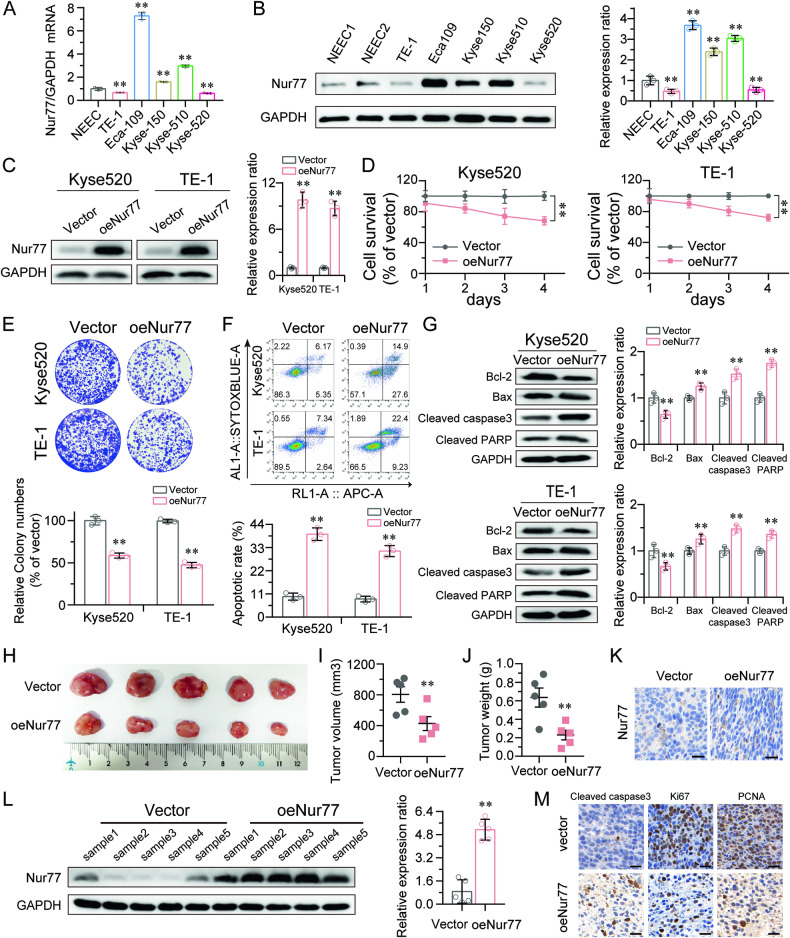
Nur77 inhibited ESCC cell proliferation in vitro and tumorigenesis in vivo. **A**, **B** qRT-PCR and western blot analysis of Nur77 expression in NEEC and ESCC cell lines (*n* = 3). **C** Overexpression of Nur77 in Kyse520 and TE-1 cell lines was analyzed by western blot. GAPDH was used as a loading control (*n* = 3). **D** The viability of ESCC cells overexpressing Nur77 was inhibited, as determined by the CCK-8 assay (*n* = 3). **E** The colony formation ability of ESCC cells overexpressing Nur77 was reduced (*n* = 3). **F** The percentage of Kyse520 and TE-1 cells that underwent Nur77 overexpression was increased (*n* = 3). **G** Western blotting was performed to investigate the expression levels of Bcl-2, Bax, cleaved caspase-3, and cleaved PARP in Kyse520 and TE-1 cells overexpressing Nur77. GAPDH was used as a loading control (*n* = 3). Representative tumor images (**H**), tumor volumes (**I**) and tumor weights (**J**) were collected from nude mice with tumor xenografts derived from TE-1 cells stably overexpressing Nur77 (*n* = 5). Nur77 protein expression in tissue samples from the xenografts was detected via immunohistochemistry (**K**) and western blotting (**L**) (*n* = 3). **M** Expression of cleaved caspase-3, Ki67, and PCNA in tumor xenografts was determined via immunohistochemistry (*n* = 3). Scale bars: 20 μm. All in vitro experiments were performed with three independent experiments. An unpaired Student’s *t* tests were used for statistical analysis, and the error bars indicate the means ± S.D. **P < 0.01 indicates a significant difference from the vector group.

To examine the biological effects of Nur77 in ESCC cells, the mRNA and protein levels of Nur77 in ESCC cell lines and primary NEEC cells were analyzed via qRT-PCR and western blotting, respectively (Fig. 1A, B). The endogenous Nur77 expression levels were lower in the Kyse520 and TE-1 cell lines, it was moderate in the Kyse150 cell line. Based on these three ESCC cell lines, cells stably overexpressing Nur77 were established (Fig. 1C and Fig. S1A), and these cells were used in subsequent functional investigations. Nur77 overexpression was shown to suppress ESCC cell growth in vitro, as demonstrated by the CCK-8 and in vitro colony formation assays (Fig. 1D, E and Fig. S1B, C). The percentage of apoptotic cells was shown to increase with increasing Nur77 overexpression, as determined by flow cytometry (Fig. 1F and Fig. S1D). Next, we measured the levels of proteins linked to apoptosis in TE-1, Kyse520 and Kyse150 cells. According to our findings, Bcl-2 was downregulated, and Bax, cleaved caspase-3 and cleaved PARP were increased in cells overexpressing Nur77 (Fig. 1G and Fig. S1E).

We next wanted to determine whether Nur77 could have a suppressive impact on the growth of tumors in living beings. Immunodeficient nude mice were subcutaneously injected with TE-1 and Kyse150 cells expressing Nur77, while a control group of mice received injections of an empty pWPXL vector. The outcomes showed that, compared with those in vector-control tumors, the tumors produced by Nur77-transduced ESCC cells were smaller and had lower tumor weights (Fig. 1H–J and Fig. S1F–H). Moreover, the xenograft tissues that overexpressed Nur77 continued to exhibit increased Nur77 expression (Fig. 1K, L). The decreases in Ki67 and PCNA expression and increase in cleaved caspase-3 expression in the Nur77-overexpressing xenografts further supported the tumor inhibitory effect of Nur77 (Fig. 1M). These findings are consistent with the in vitro results, suggesting that Nur77 may inhibit cell proliferation in vivo.

### Silencing Nur77 promotes tumor cell proliferation in vitro and in vivo

**Fig. 2:**
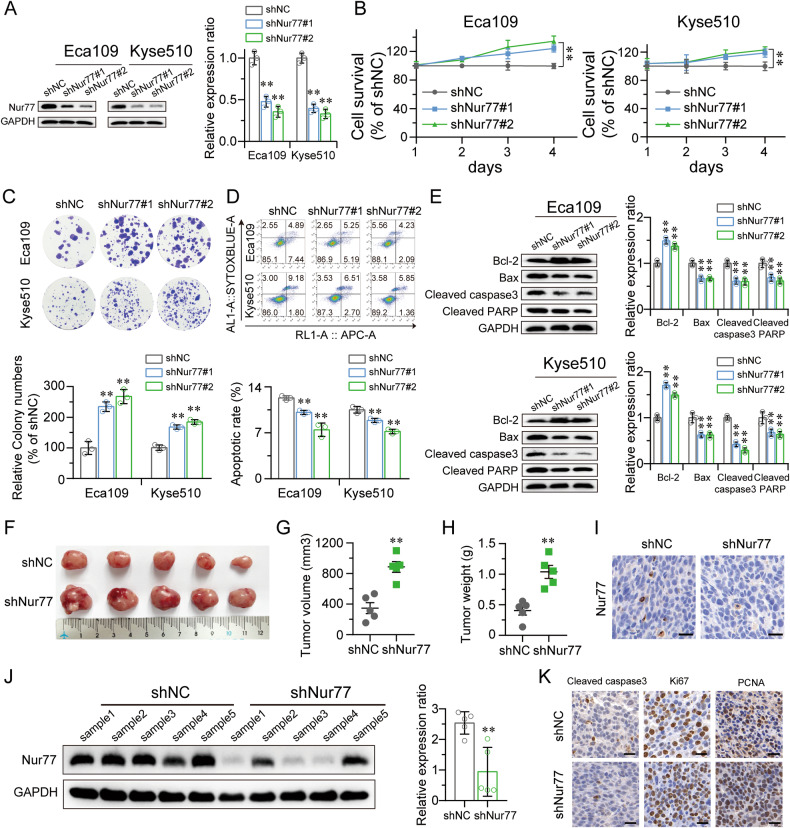
Silencing of Nur77 promoted tumor cell proliferation in vitro and in vivo. **A** Verification of Nur77 knockdown in Eca109 and Kyse510 cells by western blot analysis. GAPDH was used as a loading control (*n* = 3). Nur77 knockdown increased cell proliferation (**B**) and colony formation ability (**C**) (*n* = 3). **D** The percentage of Eca109 and Kyse510 cells undergoing apoptosis following Nur77 knockdown was decreased (*n* = 3). **E** Nur77 knockdown increased the expression of Bcl-2 and decreased the expression of Bax, cleaved caspase-3, and cleaved PARP. Cell lysates were assessed by western blotting (*n* = 3). **F** Images of tumor tissues collected from nude mice with stable Nur77 knockdown xenograft tumors derived from TE-1 cells (*n* = 5). Xenograft tumor growth was monitored (**G**) and weighed (**H**) (*n* = 5). Nur77 knockdown in tissue samples from the xenografts was evaluated via immunohistochemistry (**I**) and western blotting (**J**) (*n* = 3). **K** The expression of cleaved caspase-3, Ki67, and PCNA in tumor xenografts was determined by immunohistochemical analysis (*n* = 3). Scale bars: 20 μm. All in vitro experiments were performed with three independent experiments. An unpaired Student’s *t*-test was used for statistical analysis, and the error bars indicate the means ± S.D. **P < 0.01 indicates a significant difference from the shNC group.

Since Nur77 is linked to both in vitro and in vivo cell proliferation, we investigated whether the capacity of ESCC cells to proliferate would increase by knocking down Nur77. Two lentiviral vectors encoding efficient shRNAs targeting Nur77 were used to knockdown endogenous Nur77 expression in the three cell lines with high Nur77 expression (Fig. 2A and Fig. S1I), as it was previously noted that Nur77 was variably expressed in the panel of ESCC cell lines. The findings demonstrated that Eca109, Kyse510, and Kyse150 cells with Nur77 knockdown had greater higher proliferative and colony-forming capacities (Fig. 2B, C and Fig. S1G, K) and lower cell apoptosis (Fig. 2D and Fig. S1L). Silencing Nur77 resulted in an increase in Bcl-2 expression and a decrease in Bax, cleaved caspase-3, and cleaved PARP levels (Fig. 2E and Fig. S1M).

Next, we investigated whether the possibility that inhibiting Nur77 would promote carcinogenesis in vivo. Immunodeficient nude mice were subcutaneously injected with Eca109 and Kyse150 cells stably expressing Nur77, whereas mice injected with cells expressing the corresponding scrambled shRNA (shNC) vector served as the control group. The findings showed that, in comparison to those in tumors created from shRNA-vector cells, the tumors formed from Nur77-silenced cells were larger in both size and weight (Fig. 2F–H and Fig. S1N–P). Conversely, the xenograft tissues generated from cells that had undergone Nur77 knockdown continued to express Nur77 at low levels (Fig. 2I, J). IHC analysis of the tumors revealed that the Nur77-silenced tumors had a decreased number of cleaved caspase 3-positive positive cells and greater Ki67 and PCNA proliferation indices (Fig. 2K). Taken together, these findings revealed that knocking down Nur77 could reverse the consequences of Nur77 overexpression both in vitro and in vivo.

### Nur77 targets the IRF1 gene

**Fig. 3:**
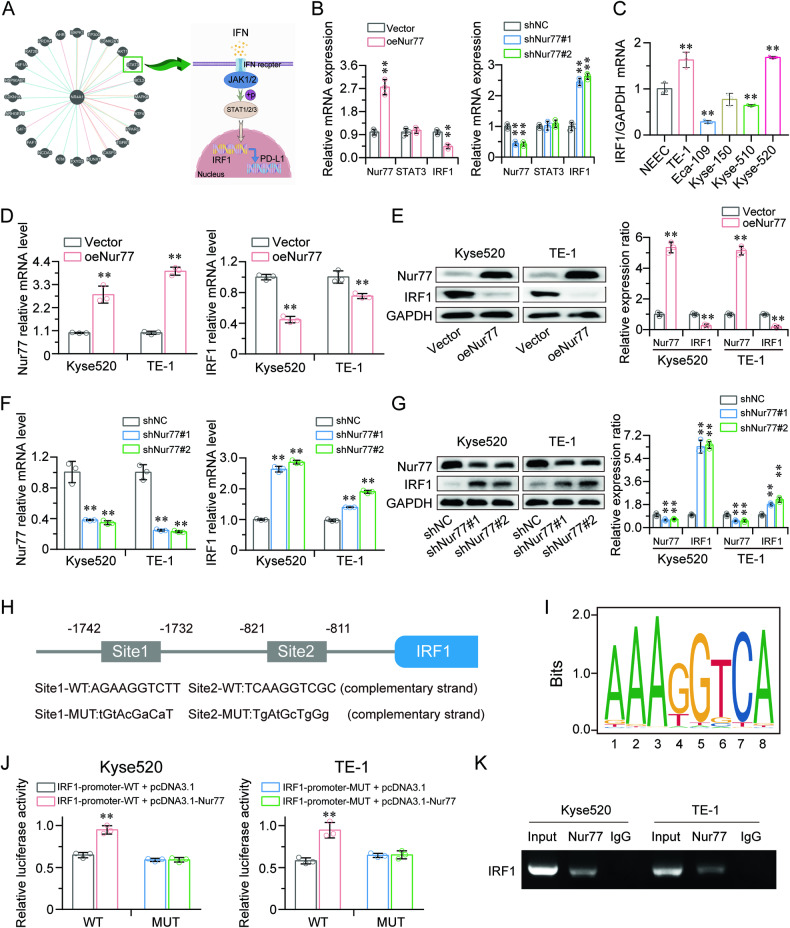
Nur77 downregulates IRF1 by directly binding to its consensus binding sequence on the IRF1 gene. **A** The Pathway Commons database was utilized to screen out target genes of Nur77, and the PPI network shows the protein‒protein interactions (left). A schematic diagram illustrates the regulation of the JAK/STAT pathway (right). **B** qRT-PCR revealed that whereas STAT3 expression remained unchanged, IRF1 mRNA was both upregulated and downregulated in Kyse520 cells overexpressing or silencing Nur77 (*n* = 3). **C** qRT‒PCR was utilized to detect IRF1 expression in ESCC cell lines (*n* = 3). qRT-PCR and western blotting showed that Kyse520 and TE-1 cells overexpressing Nur77 had decreased IRF1 mRNA (**D**) and protein (**E**) expression (*n* = 3). qRT‒PCR and western blotting showed that Kyse520 and TE-1 cells with Nur77 knockdown had increased IRF1 mRNA (**F**) and protein (**G**) expression (*n* = 3). **H** Potential Nur77 binding sites next to the transcriptional start site of the IRF1 sequence were identified via the JASPAR database (http://jaspar.genereg.net/) and via the identification of mutant sites in the IRF1 sequence. **I** The sequence logo of a potential Nur77 binding site in JASPAR. **J** Relative luciferase activities of the wild-type (WT) and mutated (MUT) IRF1 reporter plasmids in Kyse520 and TE-1 cells transfected with either Nur77 or pcDNA3.1 (*n* = 3). **K** Assessment of Nur77 binding to the IRF1 sequence in Kyse520 and TE-1 cells was performed via ChIP using an antibody against Nur77 and a negative control (IgG), which was amplified via specific primers via PCR. The data are shown as the mean ± S.D. from experiments with three replicates. An unpaired Student’s *t* test was used for statistical analysis. **P < 0.01 indicates a significant difference compared with the vehicle group.

The direct target of Nur77, which is thought to be responsible for its functional effects, was also examined. Nur77 participates in a variety of biological processes, such as apoptosis, cell proliferation, and immunological responses. To further investigate the possible antitumor mechanism of Nur77, 25 genes that interact with Nur77 were predicted from the website (http://www.pathwaycommons.org/) (Fig. 3A, left), and the STAT3 gene was selected because it is related to interferon receptor signaling pathways (Fig. 3A, right). IRF1, another transcription factor involved in the control of immunological responses, cell proliferation, and death, is encoded by the JAK/STAT signaling pathway. Then, we used qRT-PCR to determine the mRNA levels of the STAT3 and IRF1 genes. The results indicated that, in Kyse520 cells, both overexpression and knockdown of Nur77 did not alter the expression level of STAT3, but that the expression levels of IRF1 changed correspondingly (Fig. 3B). IRF1 was used in subsequent investigations because it had greater endogenous expression levels in Kyse520 and TE-1 cells (Fig. 3C) and was consistently inversely linked with Nur77 expression at both the mRNA and protein levels in both Nur77-overexpressing and Nur77-silenced cells (Fig. 3D–G).

To investigate whether Nur77 could regulate IRF1, the possible Nur77 binding region in the IRF1 sequence (Fig. 3H), which spans 931 bp (−1742~−811) close to the transcriptional start point, was examined using the JASPAR (jaspar.genereg.net) database. The pGL3 vector was modified by cloning and inserting this region. We mutated the two predicted Nur77 binding sites (AGAAGGTCTT and TCAAGGTCGC) in the IRF1 sequence (−1742 to −1732 bp and −821 to −811 bp) in accordance with the sequence logo of the potential Nur77 binding site identified by JASPAR (Fig. 3I). We discovered that the increase in luciferase activity induced by these truncated sequences significantly increased when Nur77 was overexpressed, but the increase in luciferase activity was reversed by transfection of the mutant (Fig. 3J). The ChIP results confirmed that Nur77 binds strongly to this transcriptional region of the IRF1 gene (Fig. 3K). Collectively, these findings imply that Nur77 directly targets IRF1.

### Silencing IRF1 inhibited ESCC cell proliferation, and IRF1 was involved in the suppressive effect of Nur77 on ESCC cells

**Fig. 4:**
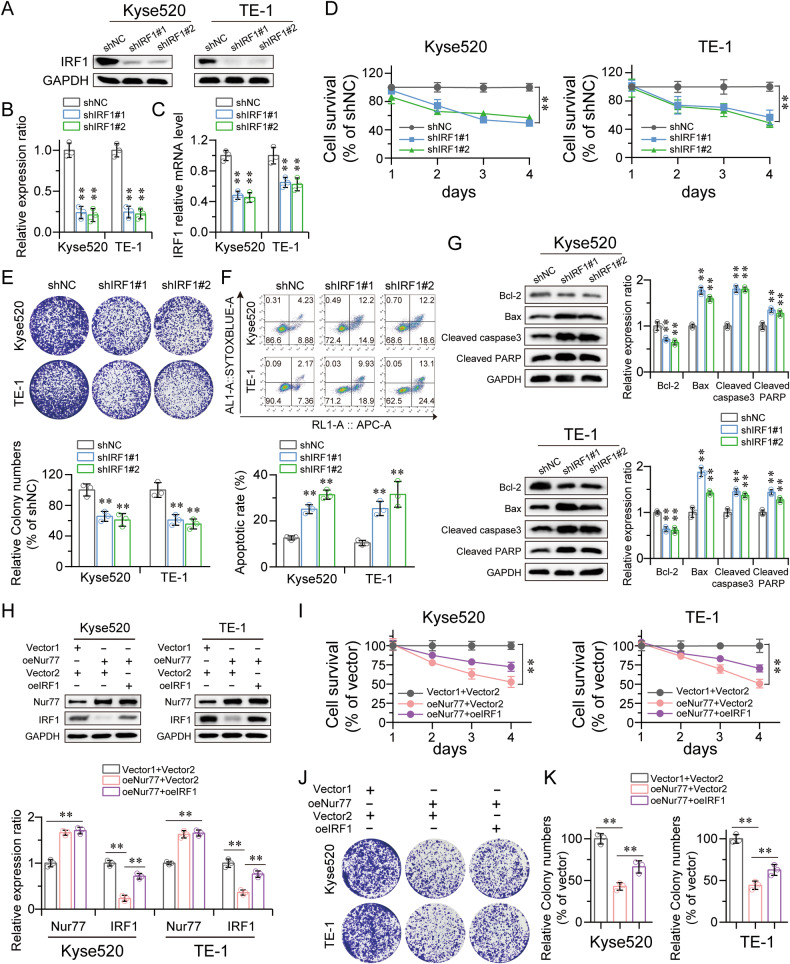
IRF1 knockdown suppressed ESCC cell proliferation, and upregulating IRF1 in ESCC cells overexpressing Nur77 rescued the suppressive effects of Nur77 in vitro. Confirmation of IRF1 knockdown in Kyse520 and TE-1 cells by western blot (**A**, **B**) and qRT‒PCR (**C**). GAPDH was used as a loading control (*n* = 3). IRF1 knockdown decreased cell proliferation (**D**) and colony formation ability (**E**) (*n* = 3). **F** The percentage of Kyse520 and TE-1 cells undergoing apoptosis following IRF1 knockdown was increased (*n* = 3). **G** Western blot analysis showed that IRF1 knockdown decreased the expression of Bcl-2 and increased the expression of Bax, cleaved caspase-3, and cleaved PARP (*n* = 3). **H** Western blot of IRF1 protein expression in Nur77-overexpressing ESCC cells overexpressing IRF1. GAPDH was used as a loading control (*n* = 3). The influence of IRF1 overexpression on the inhibitory effects of Nur77 on cell growth (**I**) and colony formation (**J**, **K**) in vitro was detected by CCK-8 and colony formation assays (*n* = 3). The data are shown as the mean ± S.D. from experiments with three replicates. An unpaired Student’s *t* test was used for statistical analysis. **P < 0.01 indicates a significant difference compared with the vehicle group.

We used Kyse520 and TE-1 cells to create stable silenced IRF1 cell lines to investigate the biological function of IRF1 in ESCC cell lines (Fig. 4A–C). The results showed that, compared with those in the pWPXL-transduced cell line, the proliferative ability of the IRF1-knockdown cells was reduced (Fig. 4D, E). Flow cytometry revealed that IRF1 knockdown increased apoptosis in Kyse520 and TE-1 cells (Fig. 4F). Apoptosis-related proteins were detected in both Kyse520 and TE-1 cells. The results demonstrated that knocking out IRF1 decreased Bcl-2 expression while increasing Bax, cleaved caspase3, and cleaved PARP levels (Fig. 4G).

Functional rescue tests were also conducted to verify that IRF1 was activated downstream of Nur77. IRF1 was upregulated in stable Nur77-overexpressing Kyse520 and TE-1 cells (Fig. 4H). In vitro CCK-8 and colony formation experiments on these cells demonstrated that overexpression of IRF1 effectively reversed the Nur77-induced suppression of cell proliferation (Fig. 4I–K). Furthermore, flow cytometry demonstrated that IRF1 overexpression successfully prevented Nur77-induced cell cycle arrest in the G2/M phase of ESCC cells (Fig. S2). The results presented above showed that IRF1 is a functional target of Nur77 and is responsible for the suppressive effects of Nur77 on ESCC cells.

### CsnB augments Nur77 tumor inhibition by downregulating IRF1 expression

**Fig. 5:**
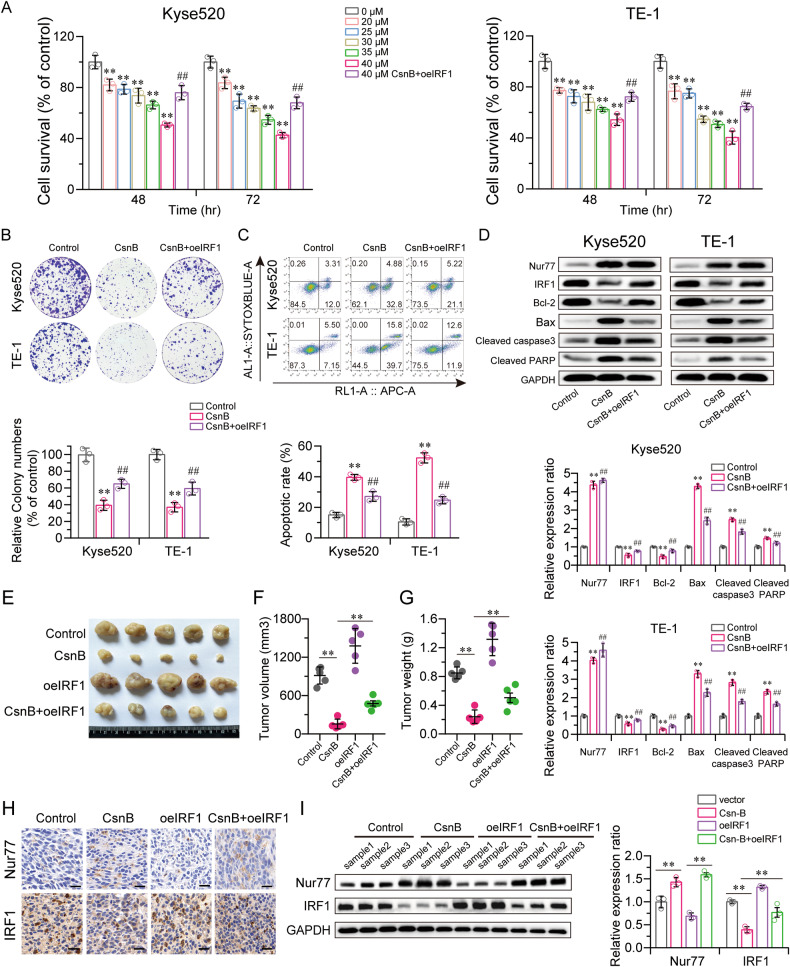
CsnB exerts its suppressive effects on ESCC cells through the downregulation of IRF1 by Nur77 in vitro and in vivo. **A** CCK-8 assays were used to measure the proliferation of Kyse520 and TE-1 cells incubated with CsnB (20, 25, 30, 35, or 40 μM) for 48 h, and overexpressing IRF1 rescued the suppressive effects of CsnB (40 μM) (*n* = 3). **B** A colony formation assay was performed to detect cell proliferation in CsnB (40 μM)-treated Kyse520 and TE-1 cells transfected with or without oeIRF1 (*n* = 3). **C** The apoptosis rate of CsnB (40 μM)-treated Kyse520 and TE-1 cells with or without oeIRF1 was detected by flow cytometry (*n* = 3). **D** Western blotting was conducted to detect the expression of Nur77, IRF1, Bcl-2, Bax, cleaved caspase 3, and cleaved PARP in 40 μM CsnB-treated Kyse520 and TE-1 cells with or without oeIRF1 (*n* = 3). **E** Typical images of tumors isolated from nude mice with tumor xenografts derived from the indicated groups (*n* = 5). Tumor volumes were monitored (**F**), and tumor weight (**G**) was measured at the time of sacrifice (*n* = 5). Nur77 and IRF1 protein expression in tissue samples from tumor xenografts treated with CsnB in the presence or absence of oeIRF1 was detected via immunohistochemistry (**H**) and western blotting (**I**) (*n* = 3). Scale bars: 20 μm. The data are shown as the mean ± S.D. from experiments with three replicates. An unpaired Student’s *t* test was used for statistical analysis. **P < 0.01 indicates a significant difference compared with the control group. ^##^*P* < 0.01 indicates a significant difference compared with the CsnB group.

CsnB is a naturally occurring Nur77 agonist. We then validated the anticancer effect of CsnB in vitro via proliferation assays and in vivo by evaluating the growth inhibition of xenograft tumors in nude mice due to the apoptotic action of CsnB via Nur77 binding. CCK-8 and colony formation experiments indicated that CsnB strongly suppressed ESCC cell proliferation, while this inhibitory effect on cancer cells could be reversed by overexpressing IRF1 in Kyse520 and TE-1 cells (Fig. 5A, B). Flow cytometry analysis revealed that IRF1 attenuated the effect of CsnB-induced cell apoptosis (Fig. 5C). We observed that the abnormal expression of Nur77 and IRF1 and apoptosis-related proteins induced by CsnB was reversed after overexpression of IRF1 in Kyse520 and TE-1 cells (Fig. 5D).

A TE-1 xenograft mouse model was generated to confirm the effects of CsnB in vivo. Treatment with CsnB reduced ESCC tumor growth, but both rapid growth and increased tumor size demonstrated that exogenous expression of IRF1 could considerably diminish the anticancer impact of CsnB in vivo (Fig. 5E–G). Furthermore, when xenograft mice were treated with CsnB in the presence or absence of IRF1, tumor tissues were prepared for IHC staining and western blotting, and the expression of Nur77 and IRF1 was evaluated (Fig. 5H, I). Overall, these findings imply that CsnB-induced IRF1 downregulation is linked to anti-ESCC effects both in vitro and in vivo.

### Targeting Nur77 synergizes with anti-PD-1 immunological checkpoint therapy

**Fig. 6:**
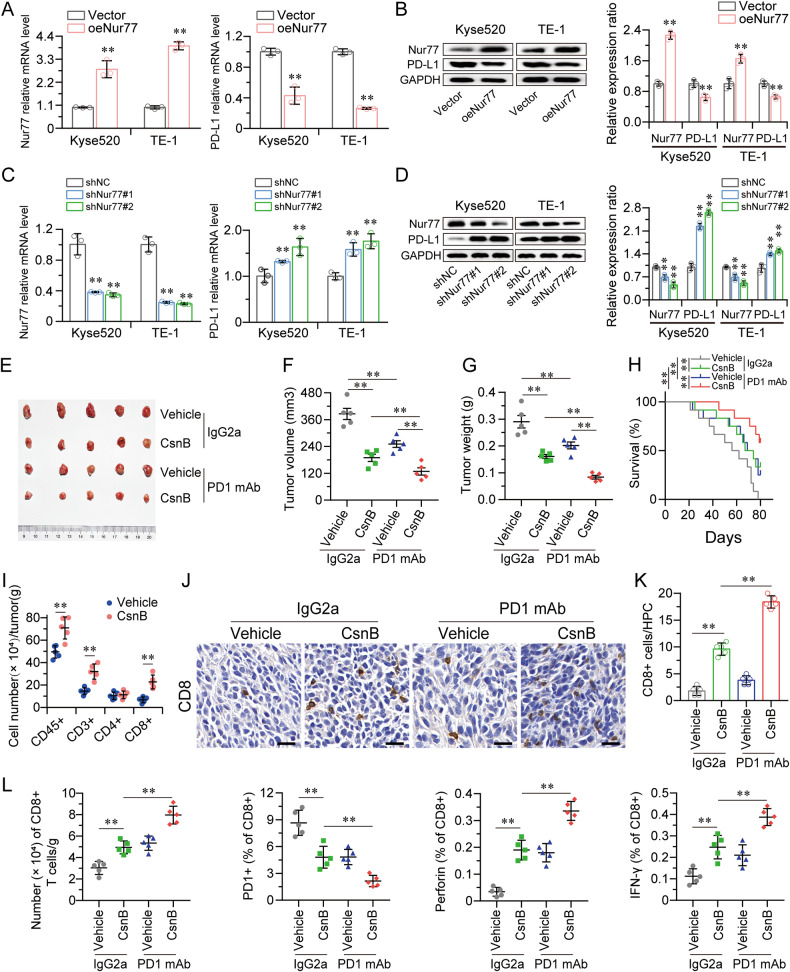
Targeting Nur77 enhances the efficacy of anti-PD-1 therapy. qRT-PCR and western blotting showed that Kyse520 and TE-1 cells overexpressing Nur77 had decreased PD-L1 mRNA (**A**) and protein (**B**) expression (*n* = 3). qRT-PCR and western blotting showed that Kyse520 and TE-1 cells with Nur77 knockdown had increased PD-L1 mRNA (**C**) and protein (**D**) expression (*n* = 3). Representative images of tumors (**E**), tumor volume (**F**), and tumor weight (**G**) of AKR xenografts from mice treated with control (vehicle) or CsnB combined with IgG2a or an anti-PD-1 mAb. Kaplan‒Meier survival curves for each group (**H**) (*n* = 5). **I** Quantification of tumor-infiltrating immune cells analyzed by flow cytometry using the indicated cell surface markers on CsnB AKR tumors grafted into C57BL/6 mice. **J**, **K** IHC staining of CD8+ T-cell infiltration in AKR xenografts treated with CsnB combined with IgG2a or an anti-PD-1 mAb. Scale bars: 20 μm (*n* = 5). **L** Quantification of CD8+, PD1+CD8+, perforin+CD8+, and IFN-γ+CD8+ cells in AKR xenografts treated with CsnB combined with IgG2a or an anti-PD-1 mAb (*n* = 5). The data are shown as the mean ± SD from experiments with three replicates. An unpaired Student’s *t* test was used for statistical analysis. **P < 0.01 indicates a significant difference compared with the vehicle group.

The promoter region of PD-L1 has two recognized IRF1 binding sites. Since PD-L1 expression is regulated by the transcription factor IRF1, we next inspected whether targeting Nur77 could increase the anticancer efficacy of PD-1 inhibition. In both the Nur77-overexpressing and Nur77-silenced cells, PD-L1 expression was consistently negatively associated with Nur77 expression at the mRNA and protein levels (Fig. 6A–D). Our findings verified that tumor development and weight were impaired in AKR model mice by CsnB therapy alone. As predicted, anti-PD-1 monotherapy also had little impact on tumor volume, tumor weight, or mouse survival. More crucially, combining Nur77 targeting with anti-PD-1 therapy increased the therapeutic benefit of these agents as compared to either monotherapy strategy (Fig. 6E–H). To determine whether the CsnB-dependent antitumor activity was related to a change in the immunological landscape, we profiled the immune cells in tumors by flow cytometry (Fig. S3). We found a comparable and significant increase in the infiltration of live CD45+ cells, CD3+ T cells, and CD8+ T cells in CsnB-treated AKR tumors as compared with vehicle-treated controls (Fig. 6I). We subsequently collected AKR tumor tissues for additional investigation. According to the IHC staining and flow cytometry analysis, greater infiltration of CD8+ T cells may be responsible for the greater responsiveness in the combination treatment groups. (Fig. 6J–L). Our findings suggest that targeting Nur77 may be a promising therapeutic strategy for improving the efficacy of anti-PD-1 therapy in ESCC patients.

### IRF1 was strongly linked to Nur77 and both were related to ESCC progression

**Fig. 7:**
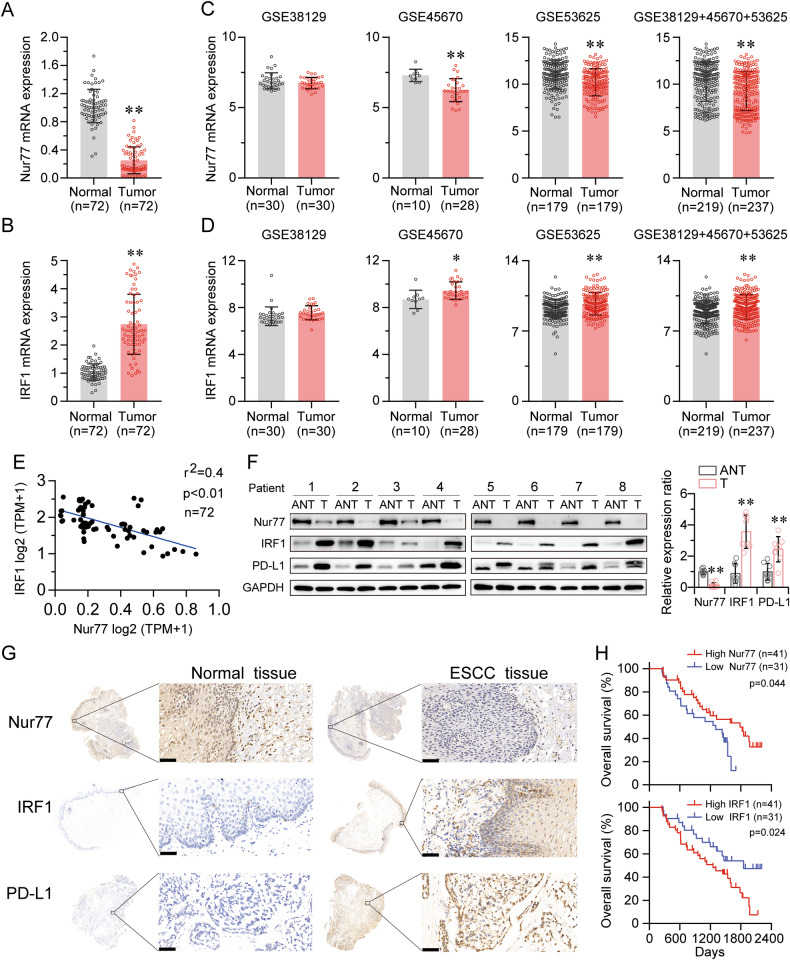
Nur77 expression was negatively correlated with IRF1 expression in primary ESCC tissues. The mRNA expression of Nur77 (**A**) and IRF1 (**B**) in 72 paired ESCC and nontumor tissues was detected via qRT‒PCR. Nur77 (**C**) and IRF1 (**D**) expression levels in ESCC and nontumor tissues in the GSE38129, GSE45670, and GSE53625 datasets. **E** The correlation between Nur77 and IRF1 mRNA expression in 72 paired cancerous/noncancerous esophageal tissues from primary ESCC patients. The Pearson correlation coefficients (r) and *p* values are indicated. **F** The protein expression levels of Nur77, IRF1, and PD-L1 in eight paired ESCC and nontumor tissues were detected via western blotting (*n* = 3). **G** Representative images of IHC staining for the Nur77, IRF1, and PD-L1 proteins in cancerous and noncancerous esophageal tissues. Scale bars: 20 μm. **H** Kaplan‒Meier analysis of overall survival stratified by Nur77 and IRF1 expression in 72 ESCC patients. The log-rank test was used to determine statistical significance. The data are shown as the mean ± S.D. from experiments with three replicates. Paired Student’s *t* tests were used for statistical analysis. *P < 0.05, **P < 0.01 indicate significant differences from the normal group.

We used qRT-PCR to assess the general mRNA expression levels of Nur77 and IRF1 in 72 pairs of human primary ESCC tissues and matched neighboring noncancerous ESCC tissues to further study their expression and connection. The results showed that Nur77 mRNA expression was dramatically downregulated, while IRF1 expression was strongly increased in ESCC tissues compared to noncancerous ESCC tissues (Fig. 7A, B); these data were consistent with those in the GSE database (Fig. 7C, D). Notably, IRF1 mRNA expression was inversely related to Nur77 expression in ESCC tissues (Fig. 7E). Western blotting and immunohistochemistry were used to confirm the expression levels of Nur77, IRF1, and PD-L1 in ESCC (Fig. 7F, G). Compared with nearby noncancerous ESCC tissues, ESCC tissues had lower Nur77 protein expression and greater IRF1 and PD-L1 protein expression. These findings also revealed a negative correlation between Nur77 and IRF1 expression. Table 1 summarizes the relationships between the Nur77/IRF1 expression level and clinicopathological features. A lower Nur77 expression level was associated with distant metastasis (*P* = 0.012) and advanced tumor/node/metastases (TNM) stage (*P* = 0.017), whereas a higher IRF1 expression was associated with lymphatic metastasis (*P* = 0.013) and advanced TNM stage (*P* = 0.029) in ESCC patients. Additionally, Kaplan‒Meier survival analysis of primary ESCC samples demonstrated that patients with higher Nur77 expression and lower IRF1 expression had longer OS than patients with lower Nur77 expression and higher IRF1 expression (Fig. 7H). These findings imply that Nur77 and IRF1 protein expression levels are inversely associated in human primary ESCC tissues and may be potential prognostic indicators for ESCC patients.

**Table 1:** The association of the expression of Nur77 and IRF1 with the clinicopathological characteristics from ESCC patients (*n* = 72).

	Nur77 expression level			IRF1 expression level		
	Low (*n* = 31)	High (*n* = 41)	*P* value	Low (*n* = 31)	High (*n* = 41)	*P* value
Age						
≤60	9	13	>0.9999	9	13	>0.9999
>60	22	28		22	28	
Sex						
Male	27	34	0.7472	25	36	0.5132
Female	4	7		6	5	
T stage						
T1 + T2	11	16	0.8096	13	14	0.6239
T3 + T4	20	25		18	27	
N stage						
N0	16	30	0.0833	25	21	0.0134
N1 + N3	15	11		6	20	
M stage						
M0	26	41	0.0121	31	36	0.0657
M1	5	0		0	5	
TNM stage						
I + II	13	29	0.0173	23	19	0.0291
III + IV	18	12		8	22	

## Discussion

ESCC is one of the most prevalent malignant malignancies worldwide and has a high fatality rate; nearly 50% of all new ESCC diagnoses are obtained in China. Men are twice as likely to develop ESCC as women are. The strongest risk factors include tobacco smoking, alcohol consumption, eating fewer fruits and vegetables, consuming hot beverages, and being older [38]. Dysphagia, gastrointestinal bleeding, discomfort, repeated aspiration or emesis, and inadvertent weight loss are common symptoms for the majority of patients [39]. Esophagogastroduodenoscopy (EGD) and biopsy are the best methods for diagnosing ESCC. Endoscopic ultrasound (EUS) is used to accurately determine the local stage, whereas cross-sectional imaging using CT, PET, or MRI provides estimates of distant metastases. Surgery, chemotherapy, radiation, molecular targeted therapy, and a combination of these strategies are currently available for ESCC treatment and are mostly dependent on the World Health Organization’s Cancer TNM staging system. However, the prognosis for ESCC patients remains poor even after receiving the most aggressive medical and surgical treatment due to the high risk of recurrence, early metastasis, and extremely low 5-year survival rate, especially in the advanced stages of the disease. Consequently, identifying novel biomarkers is critical for facilitating the early detection of ESCC and increasing patient survival rates, which will improve patient quality of life.

Among various tumor types, such as melanoma, colorectal cancer, pancreatic cancer, bladder cancer, breast cancer, ovarian cancer, gastric cancer, hepatocellular cancer, and acute myeloid leukemia, Nur77 is thought to play various roles in regulating cell growth, apoptosis, and metastasis [13–27]. Currently, little is known about the functions and processes of Nur77 in ESCC. The present findings emphasize the critical roles of Nur77 in ESCC. In this study, we demonstrated that Nur77 could inhibit the tumorigenesis and progression of ESCC via the downregulation of IRF1/PD-L1 expression and significantly enhance immunotherapy efficacy in ESCC patients. Clinical sample analysis confirmed the opposing functions of Nur77 and IRF1 in predicting ESCC patient prognosis. To our knowledge, this is the first work to discover the significant mechanisms underpinning underlying the effect of Nur77 on ESCC in vitro and in vivo.

Although there is currently no known physiological ligand for Nur77, a number of small molecules, including CsnB [37] and bis-indole-derived compounds [40], have been identified as Nur77 agonists and have been shown to have antitumor effects on gastric, colorectal, and breast cancer. CsnB prevents breast cancer by interfering with the interaction between Nur77 and the peroxisome proliferator-activated receptor (PPAR) [26]. CsnB administration dramatically suppressed Wnt signaling and intestinal tumorigenesis through Nur77 activation [21]. CsnB analogs induce mitochondrial apoptosis through Nur77 and inhibit tumor growth in gastric cancer [41]. In this study, CsnB was identified as an effective lead chemical for treating ESCC. CsnB enhances Nur77-mediated tumor inhibition by downregulating IRF1 expression, and pharmacological Nur77 stimulation with CsnB significantly enhances anti-PD-1 immunotherapy efficacy in a mouse tumor model. These findings suggested that Nur77 could be a therapeutic target for ESCC. CsnB and other Nur77 activators may constitute a new class of potent and effective anticancer medicines for future preclinical and clinical trials.

Tumor cells possess a crucial feature called metabolic reprogramming, which enables them to obtain the energy and other biological elements needed for the rapid growth and survival of cancer cells [42]. The metabolism of amino acids, fatty acids, nucleic acids, and glutamines comprises metabolic pathways [43]. Growing amounts of data indicate that Nur77 is involved in numerous metabolic processes in malignancies, including fatty acid synthesis, glutamine metabolism, and glycolysis regulation. By inhibiting the rate-limiting enzyme phosphoenolpyruvate carboxykinase (PEPCK1) sumoylation and diverting glucose metabolism toward gluconeogenesis, Nur77 inhibited the development of hepatocellular carcinoma by causing ATP depletion and stopping cell proliferation [29]. By suppressing Nur77 in pancreatic cancer cells, FBW7 (F-box and WD repeat domain-containing 7) reduces the expression of stearoyl-CoA desaturase (SCD1) and enhances ferroptosis [44]. In two distinct mouse tumor models, Nur77 inhibited the growth of breast cancer cells by preventing lipid absorption [26]. Nur77 is involved in glutamine metabolism in rhabdomyosarcoma by inhibiting the β-catenin/Myc signaling pathway, which results in a decrease in β-catenin and c-Myc levels [45]. The present study indicated that Nur77 suppressed the growth of ESCC cells and tumors by downregulating the expression of IRF1/PD-L1. However, whether Nur77 is involved in the metabolism of malignancies is unclear. However, further studies are needed to verify the specific metabolic pathway through which Nur77 inhibits tumor growth in ESCC.

IRF1, the first identified member of the IRF family, can be efficiently activated by IFN-γ, binds to promoter regions of IFN-γ-inducible genes and stimulates their transcription causing cell proliferation and apoptosis [46]. IRF1 has been shown to function as an antitumor agent in the following cancers: colorectal, pancreatic, hepatocellular, cervical, and breast cancer. IRF1 expression induces breast cancer cell specific growth suppression by inhibiting NF-κB activity [47]. In hepatocellular carcinoma, inhibition of checkpoint kinase 1 (CHK1) increases IRF1 expression to induce apoptosis and trigger antitumor immunity through major histocompatibility complex (MHC) class I-associated chain A (MICA) [48]. Activation of the STAT3/IRF1 pathway increases the susceptibility of cervical cancer cells to chemical treatments [49]. IRF1 slows the growth of pancreatic cancer by preventing the synthesis of fatty acids and mitochondrial respiration [50]. IRF1 prevents colorectal cancer by regulating pyroptosis, apoptosis, and necroptosis [51]. Conversely, IRF1 was also found to be a tumor promoter in colon adenocarcinoma, melanoma, and endometrial cancer [52, 53]. Furthermore, prior research has shown that IRF1 influences the immunological response. Because it can bind to the PD-L1 promoter, IRF1 is an essential transcription factor needed to activate PD-L1 production. Aberrant expression of IRF1 is sufficient to induce PD-L1 upregulation even in the absence of IFN-γ [54]. IRF1 inhibited antitumor immunity by upregulating PD-L1 in colon adenocarcinoma and melanoma cells [52]. IRF1 is essential for tumor immune evasion caused by SPOP mutations in endometrial cancer [53]. In hepatocellular carcinoma cells, nuclear interleukin-33 (IL-33) SUMOylation stabilizes IRF1 to aid in immunological evasion [55]. These investigations showed that IRF1 is a dual-edged sword that can increase cancer immune evasion by upregulating PD-L1 and promoting antitumor effects. However, the relationships between Nur77 and IRF1 and their specific functions in ESCC remain poorly understood. The results of the present investigation demonstrated that Nur77 directly regulates IRF1, and that IRF1 knockdown accelerates apoptosis and reduces proliferation in ESCC cells. Moreover, rescue trials demonstrated that IRF1 knockdown may significantly boost the antiproliferative effect of Nur77 downregulation, indicating that IRF1 is required for the proapoptotic and antiproliferative effects of Nur77. Furthermore, compared to that of either monotherapy, the combination of the Nur77 agonist CsnB and anti-PD-1 therapy greatly improved the therapeutic efficacy of these agents. These findings suggested that Nur77 controls PD-L1 expression through IRF1 and influences immunotherapy through checkpoint blockade. However, the specific mechanisms through which Nur77 mediates antitumor immunity require further investigation.

## Conclusions

In summary, our findings show that Nur77 plays important anticancer roles through direct impacts on ESCC cells and boosts antitumor immunity by downregulating the expression of IRF1 and PD-L1. This is the first study to investigate the functions and processes of Nur77 and IRF1 in ESCC. This research raises the possibility that Nur77 and/or IRF1 could be used as separate prognostic biomarkers or therapeutic targets for ESCC.

## Materials and methods

### Cell lines and cell culture

The normal esophageal epithelial cell (NEEC) and human ESCC cell lines Eca109, Kyse150, Kyse510, Kyse520, and TE1 were kindly provided by Shanghai Whelab Bioscience Limited. The murine ESCC cell line AKR was purchased from Shanghai Yaji Biotechnology Co., Ltd. NECC were cultured in high-glucose minimum essential medium (HyClone). Eca109, Kyse150, and TE1 cells were cultured in RPMI 1640 medium (HyClone, Logan, UT). Kyse510 and Kyse520 cells were cultured in 50% RPMI 1640 mixed medium and 50% F12 medium (HyClone). All of the aforementioned media were kept at 37 °C in a humidified incubator with 5% CO_2_ and contained 10% fetal bovine serum (FBS; Yeasen Biologic Technology Co., Ltd, Shanghai, China) and 1% penicillin/streptomycin (New Cell & Molecular Biotech Co., Ltd., Suzhou, China). The vendors of the cells verified and described each one.

### Reagents

CsnB was purchased from TargetMol (Cat# T3976, USA) and dissolved in DMSO. Both the InVivoMab rat IgG2a isotype control (BE0089) and the InVivoMab anti-mouse PD-1 (CD279) (BE0273) were obtained from Bio X cells and diluted in InVivoPure pH 7.0 dilution buffer (IP0070).

### Lentivirus infection

A lentiviral expression system was used to assess whether Nur77 and IRF1 were knocked down or overexpressed. Lentiviral vectors obtained from GeneChem Co. (Shanghai, China), were used to transfect ESCC cells (3 × 10^5^) in accordance with the manufacturer’s instructions. The medium was then changed to complete medium after 16 h. Stable cell lines expressing Nur77 or Nur77 shRNA or IRF1 or IRF1 shRNA were established for 3 weeks using 0.5 μg/mL puromycin 48 h after infection. qRT-PCR and western blotting were used to evaluate the effectiveness of the overexpression and knockdown agents, respectively, at 48 h after transfection. The following is a list of the target primer sequences: shNC: TTCTCCGAACGTGTCACGT; shNur77-1: GCCCAGCTTCAGCACCTTCAT; shNur77-2: CAGCACTGCCAAACTGGACTA; shNur77-3: ACCCATCATTGACAAGATCTT; shIRF1-1: GCGTGTCTTCACAGATCTGAA; shIRF1-2: AGATGCTAAGAGCAAGGCCAA, and shIRF1-3: CGTGTGGATCTTGCCACATTT.

### Quantitative real-time polymerase chain reaction (qRT-PCR)

Total RNA was extracted from 72 pairs of human primary ESCC tissues and cell lines using TRIzol reagent (Invitrogen, USA). A reverse transcription kit (Yeasen Biotech Co. Ltd, Shanghai, China) was then used to convert the RNA into cDNA. With an ABI 7500 system (Biosystems, Foster City, CA, USA), the mRNA expression levels of the target genes were standardized to those of the housekeeping gene GAPDH through the use of qRT‒PCR and SYBR Green PCR master mix reagents (Takara, Japan). We used the comparative CT (2^−ΔΔCT^) method to evaluate the qRT‒PCR results. The following primer sequences (5′-3′) were used to quantify the target genes:

Nur77-F: AGAGTTTGACACCTTCCTCTAC

Nur77-R: GAAGTCCTCGAACTTGAAGGAG

IRF1-F: AAGGGGTGTGGCCTTTTTAGA

IRF1-R: TGTCCCTGTTCACCCCAAAG

CD274-F: TGGCATTTGCTGAACGCATTT

CD274-R: TGCAGCCAGGTCTAATTGTTTT

GAPDH-F: AGAAGGCTGGGGCTCATTTG

GAPDH-R: AGGGGCCATCCACAGTCTTC

### Cell proliferation assay

Infected and ESCC cells were plated in 96-well plates (4000 cells/well) and ESCC cells were treated with gradient doses of CsnB for 48 h to assess proliferation via the Cell Counting Kit-8 (CCK-8; Life-iLab, Shanghai, China) assay. The absorbance was determined with a microplate reader (Biotek, Winooski, VT) set at 450 nm.

### Colony formation assay

In 6-well plates with 2000 cells/well, both infected and ESCC cells were plated. The ESCC cells were then treated with gradient doses of CsnB for 48 h. Colonies were fixed with 4% paraformaldehyde and stained with 0.1% crystal violet solution (Servicebio, Wuhan, China) for 20 min after 2–3 weeks. Colonies per well were counted using the ImageJ program.

### Flow cytometry assay and tumor immune phenotyping

As directed by the manufacturer, apoptotic cells were visualized using an Annexin V-FITC/propidium iodide (PI) double staining kit (MultiSciences Biotech Co. Ltd., Hangzhou, China). Briefly, 3 × 10^5^ cells/well of both infected and ESCC cells treated with CsnB were plated in 6-well culture trays. After that, the cells were removed, washed twice with cold PBS, and then resuspended in 500 μL of binding buffer supplemented with 5 μL of Annexin V-FITC and PI solution at room temperature in the dark for 30 min. The labeled cells were quantified using flow cytometry (NovoCyte; ACEA Biosciences, San Diego, CA).

To prepare a single-cell suspension, tumor tissues were removed, broken up into fragments, digested using a tumor dissociation kit (Miltenyi Biotec), and then treated as directed using a cell stimulation cocktail (00-4975-03, eBioscience). After being cleaned and resuspended in fluorescence-activated cell sorting buffer at 4 °C, the cells were stained for multicolor flow cytometry using fluorescent-conjugated antibodies and the proper isotype controls. The following antibodies were utilized for cell surface staining: CD45, CD3, CD4, CD8, and PD1. Intracellular markers such as Foxp3 and perforin were stained, and intracellular cytokine levels were measured following treatment with Cell Stimulation Cocktail (Invitrogen) and staining for IFN-γ after fixation and permeabilization. The gating approach for flow cytometry analysis of lymphoid and myeloid populations in AKR tumors is shown in Additional File 1: Fig. S3.

### Western blotting

Total proteins were extracted from cells or tissues using cell or tissue total protein extraction reagent (Boster, Wuhan, China) supplemented with protease and phosphatase inhibitors (Roche, Switzerland). The protein content was determined using a NANO-400A (Allsheng, Hangzhou, China). Equal volumes of protein were transblotted onto 0.22 μm polyvinylidene difluoride (PVDF) membranes (Merck Millipore) after being separated via electrophoresis on 10% polyacrylamide gels (Cat# PG212, EpiZyme Biotechnology, Shanghai, China). After being blocked for an hour at room temperature in 5% nonfat milk, the membranes were treated with primary antibodies against Nur77 (1:200, Cat# sc-365113, Santa), IRF1 (1:500, Cat# T55507S, Abmart), Bcl-2 (1:1000, Cat# 15071S, Cell Signaling Technology), Bax (1:2000, Cat# T40051S, Abmart), Cleaved-caspase3 (1:1000, Cat#9664S, Cell Signaling Technology), Cleaved-PARP (1:1000, Cat# T55035S, Abmart), PD-L1 (1:2000, Cat# 66248-1-lg, Proteintech), and GAPDH (1:3000, Cat# 60004-1-Ig, Proteintech) at 4 °C overnight. Afterward, the membranes were incubated with HRP-conjugated anti-rabbit IgG (1:2000, Cat# 7074S, Cell Signaling Technology) or anti-mouse IgG (1:2000, Cat# 7076S, Cell Signaling Technology) secondary antibodies for 1 h at room temperature. An LAS-3000 image analyzer and MultiGauge software (Fujifilm Corporation, Tokyo, Japan) were used to visualize the protein bands on the membranes after they had been cleaned with Tris buffered saline containing Tween 20 (TBST, Solarbio) for various exposure times. The software ImageJ was used to measure the band density.

### Immunohistochemical analysis

As previously mentioned, immunohistochemical (IHC) staining was carried out. To summarize, 4 μm thick slices of paraffin-embedded tumor samples from the mouse model experiment and clinical ESCC tissues were cut. After deparaffinization, the sections were rehydrated. Following antigen retrieval, the slices were incubated overnight at 4 °C with anti-Nur77 (1:200, Cat# NB100-56745, Novus), anti-IRF1 (1:100, Cat# 11335-1-AP, Proteintech), anti-PD-L1 (1:5000, Cat# 66248-1-lg, Proteintech), anti-Ki67 (1:300, Cat# GB121141, Servicebio) anti-PCNA (1:1000, Cat# GB12010, Servicebio) and anti-CD8 (1:400, Cat# GB15068, Servicebio) antibodies. Following multiple washes, the sections were incubated for 1 h at room temperature with an HRP-conjugated secondary antibody and stained with 3,3′-diaminobenzidine tetrahydrochloride (DAB, Servicebio, Wuhan, China). The slides were imaged using a microscope (Olympus BX43F, Japan), and the images were processed using CaseViewer software.

### Animal experiments

Male BALB/c nude mice and BALB/c mice (4~5 weeks) were purchased from Shanghai Model Organisms Center, Inc. (Shanghai, China). For the immunodeficient mouse model, ~5 × 10^6^ shNC, shNur77, vector, oeNur77, or wild-type TE-1 cells were subcutaneously injected into the right dorsal flank of BABL/c nude mice. For the immunocompetent mouse model, 4 × 10^6^ AKRs were injected subcutaneously into BALB/c mice. When tumor grown to a volume of about 50 mm^3^, mice were randomly divided into different groups (5 mice/group).The number of mice in each group for each experiment is indicated in the figure legends. Twice a week, tumor growth was measured using a sliding caliper, and tumor volume was calculated using the formula: length × width^2^ × 0.5. Mice were euthanized 4 weeks later and the tumor tissues were removed for weight measurement and further IHC staining.

For Nur77 agonists (CsnB) treatments, mice were given CsnB (13 mg/kg) intraperitoneally twice a week once the tumor size reached 50 mm^3^. For anti-PD1 treatments, mice were intraperitoneally injected with mouse anti-PD1 antibodies (100 µg per animal) or an IgG isotype control (Bio X Cell) every 3 days for 2 weeks beginning when the tumor size reached 50 mm^3^. Tumors were collected at the end of the trial and placed in tissue preservation fluid (Cat# abs9474, Absin) for further tumor immune phenotyping and flow cytometry analysis performed by Universal Biotech Co., Ltd. (Shanghai, China). In this experiment, blinding is not used. At least three independent experiments were performed. All experimental procedures were approved by Department of Laboratory Animal Science Fudan University with the approved number was 2021JS Huashan Hospital-452.

### Dual-luciferase reporter assays

The JASPAR core (http://jaspar.genereg.net/) was used to predict putative binding sites for Nur77 on the 3′-UTR of IRF1, which ranged from −2000 to +200 of the TSS. DNA segments of both the wild-type and mutant types were cloned and inserted into the pGL3-Basic vector (Promega). TE-1 and Kyse520 cells (2 × 10^4^ cells/well) were cotransfected with the corresponding plasmids after being plated in a 24-well plate. Firefly and Renilla luciferase activities were measured using the Dual-Luciferase® Reporter Assay System (Promega, USA) in accordance with the manufacturer’s procedure.

### Chromatin immunoprecipitation (ChIP) assays

A Thermo Fisher Scientific ChIP Assay Kit (Cat# 26157) was used to perform ChIP assays in Kyse520 and TE-1 cells in accordance with the manufacturer’s instructions. Then, 10% formaldehyde was used to cross-link the cells, and 1 M glycine was used to quench the proteins. Following a 1× PBS wash, the cells were centrifuged for 5 min at 2000 rpm and then treated with Tissue Protein Extraction Reagent (Thermo Scientific) for 5 min in an ice bath. The sediments were submerged in nuclear lysis buffer, and sonication was used to separate the DNA into fragments ranging from 150 to 900 bp in length. Using protein A/G magnetic beads (Cat# 88802, Thermo Fisher Scientific) and antibodies against Nur77 (1:200, Cat# NB100-56745, Novus) or control rabbit IgG (1:100, Cat# 2729, Cell Signaling Technology), the nuclear lysate was incubated at 4 °C overnight on a rotator. The DNA was extracted and subjected to PCR amplification using the following primers after the crosslinking reactions were reversed:

IRF1-ChIP-F: GGATTAATAAAGAGG

IRF1-ChIP-R: TAATTTCCCTTCCTC

### Cell cycle analysis

Following transfection with either Nur77 or IRF1, the cells were harvested, fixed in 50% ethanol, treated with 5 mg/ml RNase A (Bioneer, Daedeok-gu, Daejeon, Korea), stained with 50 μg/ml propidium iodide, and subjected to flow cytometry analysis (Partec, Germany, using FlowJo software) to determine characteristics of the cell cycle.

### Patient samples

Seventy-two patients with ESCC underwent esophageal surgery at Huashan Hospital, Fudan University (Shanghai, China) between November 2016 and April 2023. Tumor tissues and pair-matched normal tissues were acquired from these patients. All surgical clinical samples were frozen and stored in liquid nitrogen until needed. Eight ESCC tissues and matched adjacent noncancerous esophageal tissues were subjected to quantitative western blot analysis. Prior to receiving chemical and radiation therapy, all patient tissues were removed. All ESCC tissues were obtained from patients only after written informed consent was obtained. The World Health Organization’s pathologic categorization system was used to evaluate the histological classification. The 8th edition of the AJCC TNM staging guide was used to evaluate the differentiation grade and TNM stage. The Huashan Hospital Institutional Review Board granted approval for this study under approval number KY2020-837.

### Gene expression data analysis

The NCBI GEO database gene expression data (accessions numbers GSE38129, GSE45670, and GSE53625) are openly accessible. These data were downloaded for additional analysis utilizing BRB array tools.

### Statistical analysis

All the data are expressed as the mean ± standard deviation (S.D.) of at least three independent experiments. For statistical analysis, SPSS 22.0 and GraphPad Prism 7.0 were utilized. The in vitro and in vivo data were analyzed via the Student’s *t* test followed by Dunnett’s post hoc test. The correlation coefficient between Nur77 and IRF1 expression was determined using Spearman’s analysis. The expression of Nur77 and IRF1 mRNA in cancer tissues was compared to that in adjacent normal tissues using the Wilcoxon signed ranks test. Correlations between Nur77 and IRF1 expression and clinicopathological features were evaluated using Fisher’s exact test. Survival status was evaluated utilizing the log-rank test and the Kaplan‒Meier method. All statistical analyses were two-sided. Statistical significance was defined as *P < 0.05, **P < 0.01, and ^##^*P* < 0.01.

### Supplementary information

Supplementary Material

Original Western Blots

## Data Availability

The data can be obtained from the corresponding authors upon request.
