# Supplementary material for: Nur77-IRF1 axis inhibits esophageal squamous cell carcinoma growth and improves anti-PD-1 treatment efficacy
Source: Cell Death Discov. 2024 May 24;10:254. doi: 10.1038/s41420-024-02019-x (PMC11126585; doi:10.1038/s41420-024-02019-x)
Supplement: Supplementary file 1 — Supplementary Material [file 41420_2024_2019_MOESM1_ESM.docx]

**Supplementary Material**

**Nur77-IRF1 axis inhibits esophageal squamous cell carcinoma growth and improves anti-PD-1 treatment efficacy**

Huanying Shi^1#^, Lu Chen^1#^, Tianxiao Wang^1^, Wenxin Zhang^1^, Jiafeng Liu^1^, Yuxin Huang^1^, Jiyifan Li^1^, Huijie Qi^1^, Zimei Wu^1^, Yi Wang^1^, Haifei Chen^1*^, Yongjun Zhu^2*^, Qunyi Li^1*^

1 Department of Pharmacy, Huashan Hospital, Fudan University, No.12 Urumqi Middle Road, Shanghai, 200040, China.

2 Department of Cardio-Thoracic Surgery, Huashan Hospital, Fudan University, No.12 Urumqi Middle Road, Shanghai, 200040, China.

*Corresponding author:

Qunyi Li or Haifei Chen, Department of Pharmacy, Huashan Hospital, Fudan University, No.12 Urumqi Middle Road, Shanghai, 200040, China. E-mail: qyli1234@163.com, or [frayerchen@163.com](mailto:frayerchen@163.com);

Yongjun Zhu, Department of Cardio-Thoracic Surgery, Huashan Hospital, Fudan University, No.12 Urumqi Middle Road, Shanghai, 200040, China. E-mail: zhuyongjun_md@163.com.

These authors contributed equally to this work: Huanying Shi, Lu Chen

**Competing interests**

The authors declare no conflict of interest.

**ORCID**

Qunyi Li**:** <https://orcid.org/0000-0001-5522-1562>


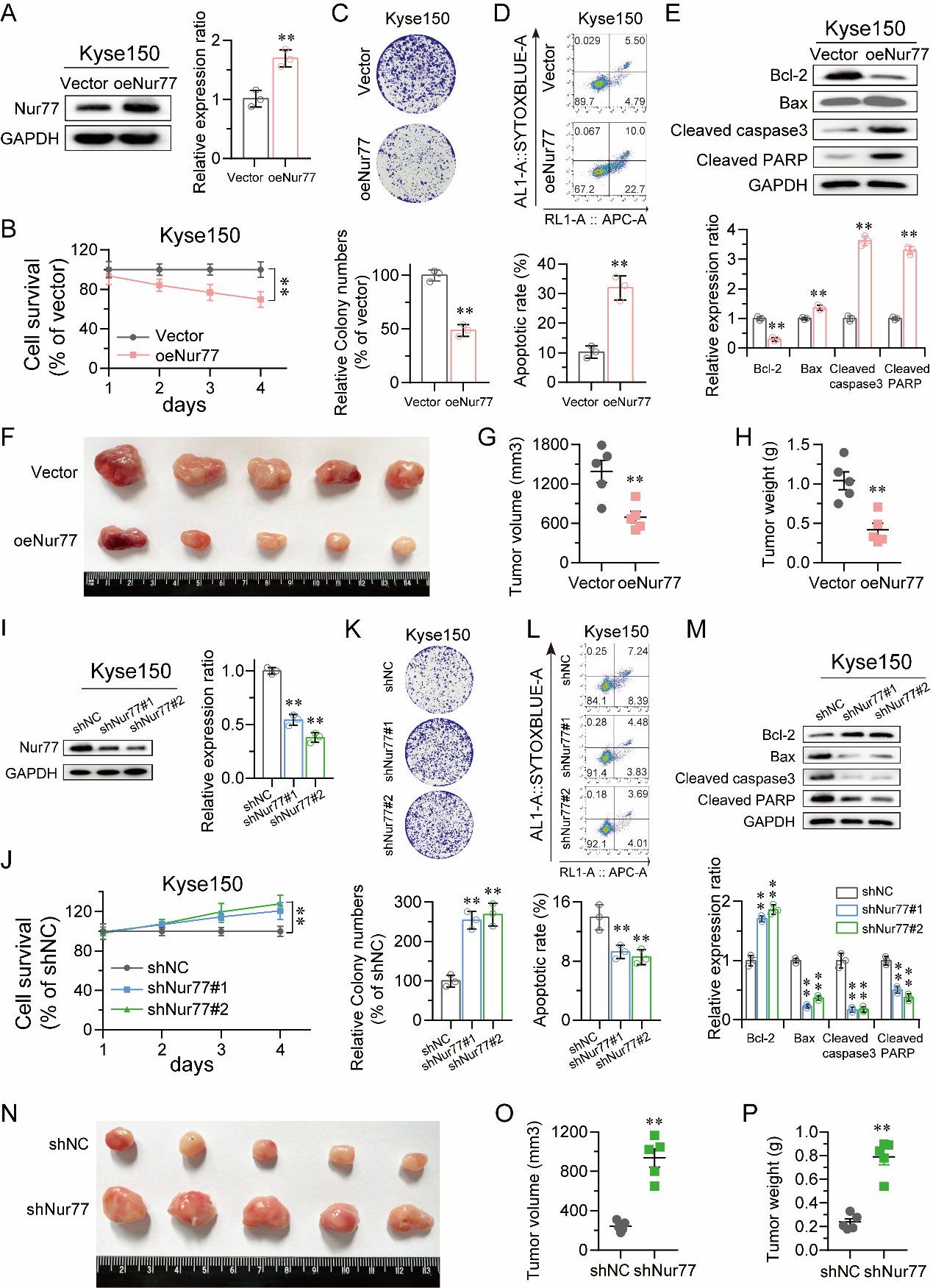


**Supplementary Fig. S1. Nur77 overexpression inhibits Kyse150 cells proliferation and promotes apoptosis, whereas Nur77 knockdown increases Kyse150 cell proliferation and suppresses apoptosis in vitro and in vivo.**

(**A**) Overexpression of Nur77 in Kyse150 cells was analyzed by western blot. GAPDH was used as a loading control. (n=3).

(**B**) The viability of ESCC cells overexpressing Nur77 was inhibited, as determined by the CCK-8 assay. (n=3).

(**C**) The colony formation ability of ESCC cells overexpressing Nur77 was reduced. (n=3).

(**D**) The percentage of Kyse150 cells that underwent Nur77 overexpression was increased. (n=3).

(**E**) Western blotting was performed to investigate the expression levels of Bcl-2, Bax, cleaved caspase-3, and cleaved PARP in Kyse150 cells overexpressing Nur77. GAPDH was used as a loading control. (n=3).

(**F-H**) Representative tumor images (F), tumor volumes (G) and tumor weights (H) were collected from nude mice with tumor xenografts derived from Kyse150 cells stably overexpressing Nur77. (n=5).

(**I**) Verification of Nur77 knockdown in Kyse150 cells by western blot analysis. GAPDH was used as a loading control. (n=3).

(**J and K**) Nur77 knockdown increased cell proliferation (J) and colony formation ability (K). (n=3).

(**L**) The percentage of Kyse150 cells undergoing apoptosis following Nur77 knockdown was decreased. (n=3).

(**M**) Nur77 knockdown increased the expression of Bcl-2 and decreased the expression of Bax, cleaved caspase-3, and cleaved PARP. Cell lysates were assessed by Western blotting. (n=3).

(**N**) Images of tumor tissues collected from nude mice with stable Nur77 knockdown xenograft tumors derived from Kyse150 cells. (n=5).

(**O and P**) Xenograft tumor growth was monitored (O) and weighed (P). (n=5).

All in vitro experiments were performed with three independent experiments. An unpaired Student’s t tests were used for statistical analysis, and the error bars indicate the means ± S.D. **P < 0.01 indicates a significant difference from the vector or shNC group.


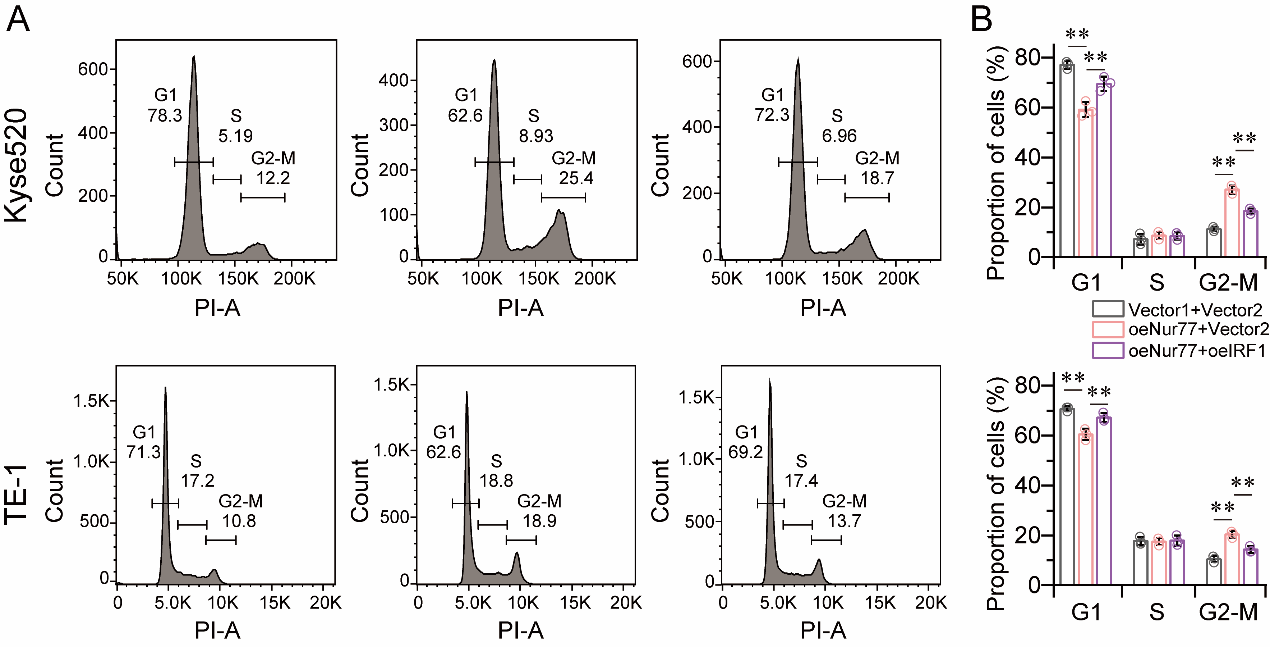


**Supplementary Fig. S2. IRF1 prevents Nur77-induced G2/M cell cycle arrest in Kyse520 and TE-1 cells.**

(**A**) Flow cytometry was performed in the Kyse520 and TE-1 cells to analyze the cell cycle following transfected with Nur77 or IRF1.

(**B**) The distribution were quantified by GraphPad software. (n = 3).

The data are shown as the mean ± S.D. from experiments with three replicates. An unpaired Student’s t test was used for statistical analysis. **P < 0.01.

**
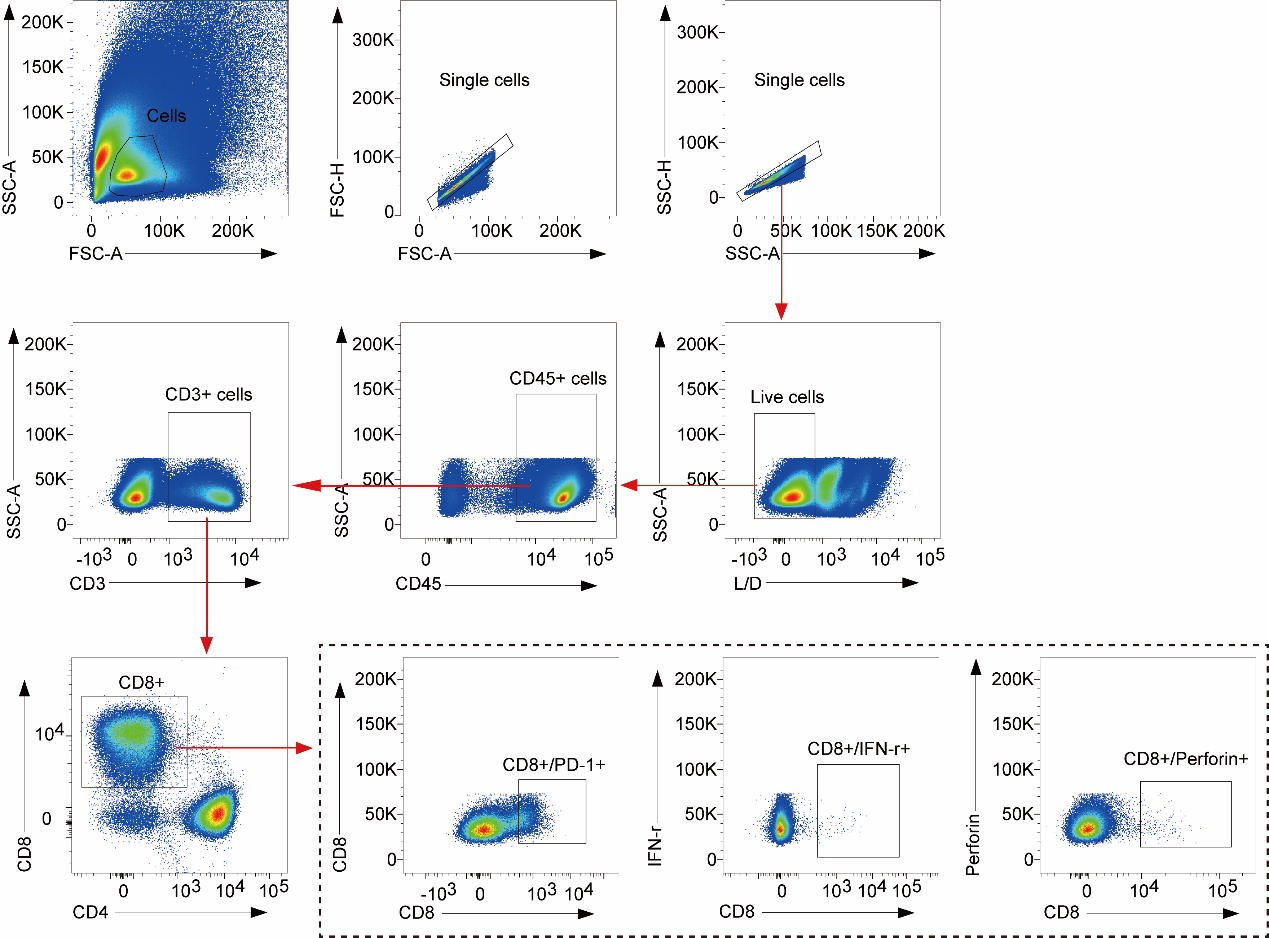
**

**Supplementary Fig. S3.** Gating strategy for flow cytometry analysis of lymphoid and myeloid population in AKR tumors.
