# Supplementary material for: Nur77-IRF1 axis inhibits esophageal squamous cell carcinoma growth and improves anti-PD-1 treatment efficacy
Source: Cell Death Discov. 2024 May 24;10:254. doi: 10.1038/s41420-024-02019-x (PMC11126585; doi:10.1038/s41420-024-02019-x)
Supplement: Supplementary file 2 — Original Western Blots [file 41420_2024_2019_MOESM2_ESM.pdf]

Original Western Blots

Figure 1

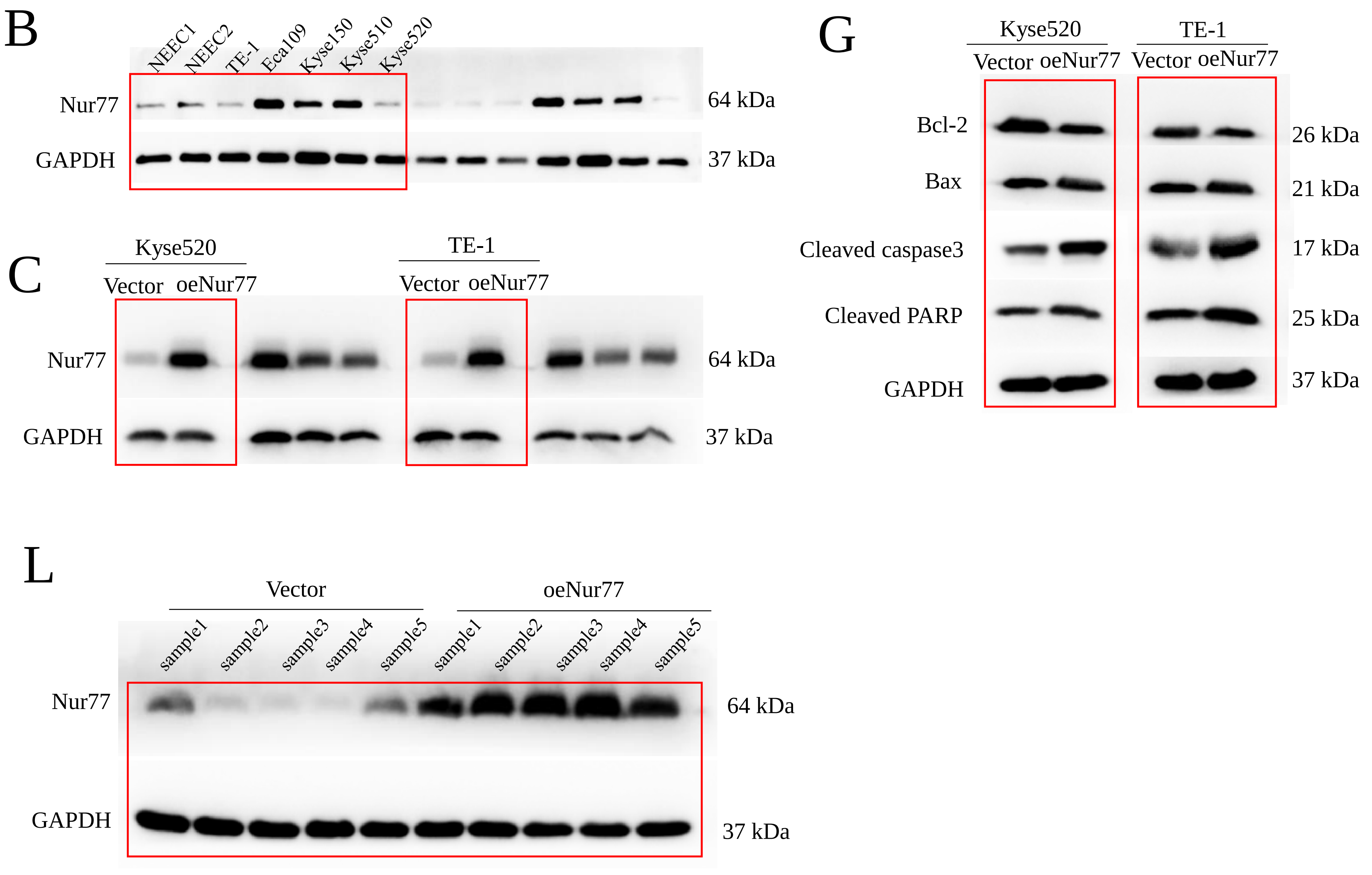
B
NEEC1
NEEC2
TE-1
Eca109
Kyse150
Kyse510
Kyse520
Nur77
GAPDH
64 kDa
37 kDa
C
Kyse520
TE-1
Vector
oeNur77
Vector
oeNur77
Nur77
GAPDH
64 kDa
37 kDa
G
Kyse520
TE-1
Vector
oeNur77
Vector
oeNur77
Bcl-2
Bax
Cleaved caspase3
Cleaved PARP
GAPDH
26 kDa
21 kDa
17 kDa
25 kDa
37 kDa
L
Vector
oeNur77
sample1
sample2
sample3
sample4
sample5
sample1
sample2
sample3
sample4
sample5
Nur77
GAPDH
64 kDa
37 kDa

Figure 2

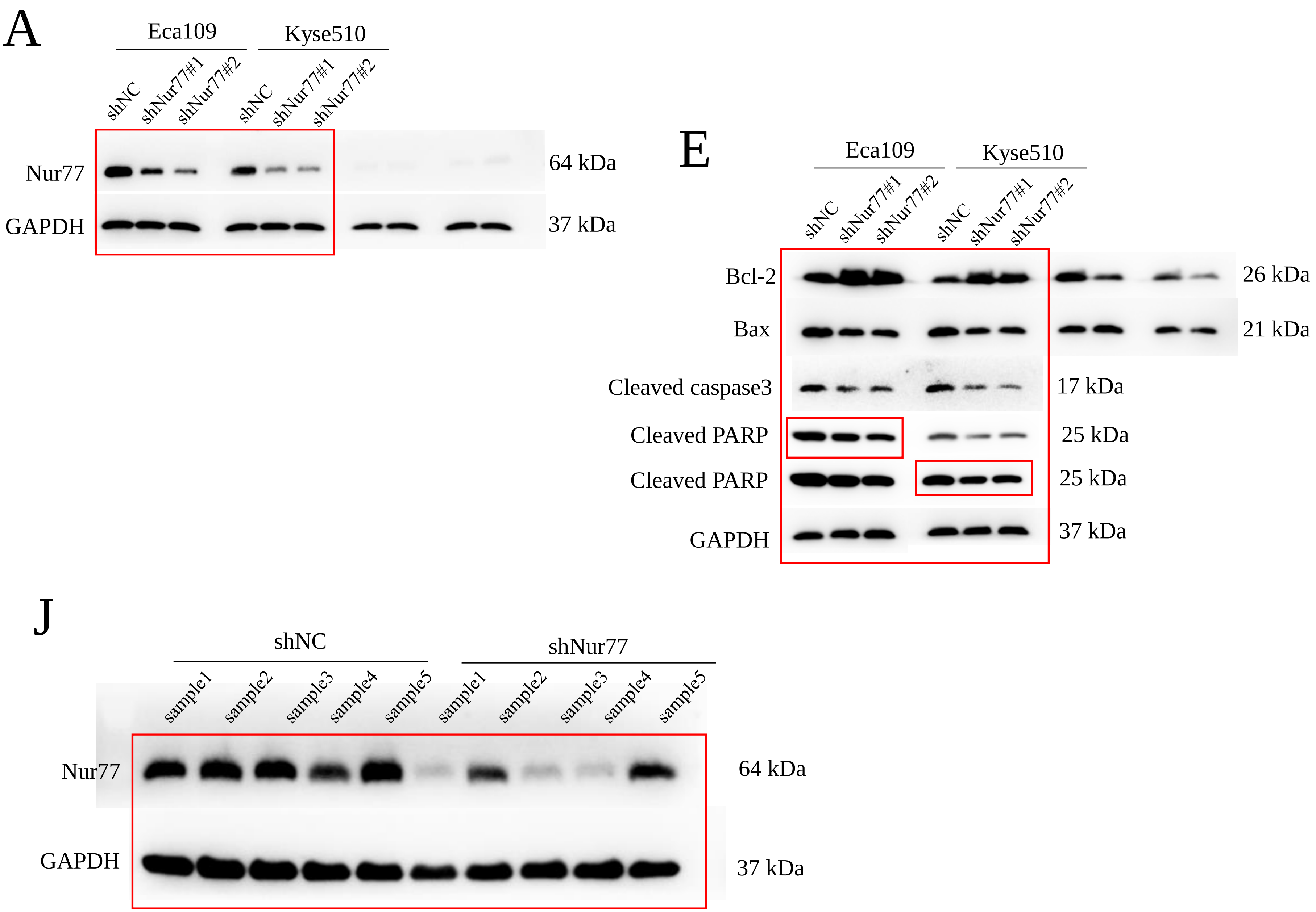
A
Eca109
Kyse510
shNC
shNur77#1
shNur77#2
Nur77
GAPDH
64 kDa
37 kDa
E
Bcl-2
Bax
Cleaved caspase3
Cleaved PARP
Cleaved PARP
26 kDa
21 kDa
17 kDa
25 kDa
25 kDa
J
shNC
shNur77
sample1
sample2
sample3
sample4
sample5

Figure 3

E

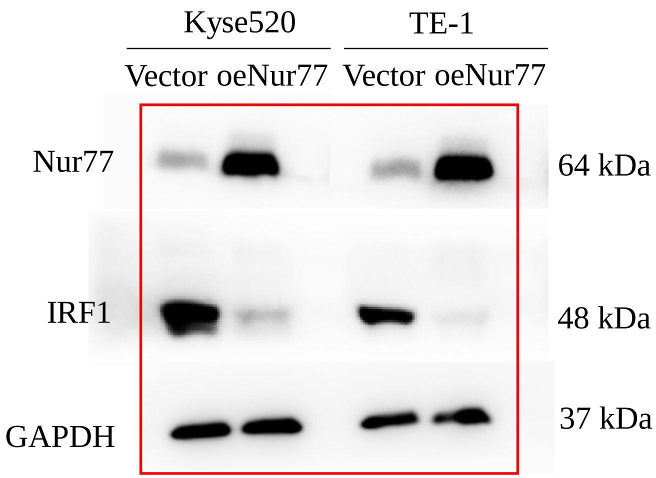
Kyse520
TE-1
Vector oeNur77 Vector oeNur77
Nur77
IRF1
GAPDH
64 kDa
48 kDa
37 kDa

G

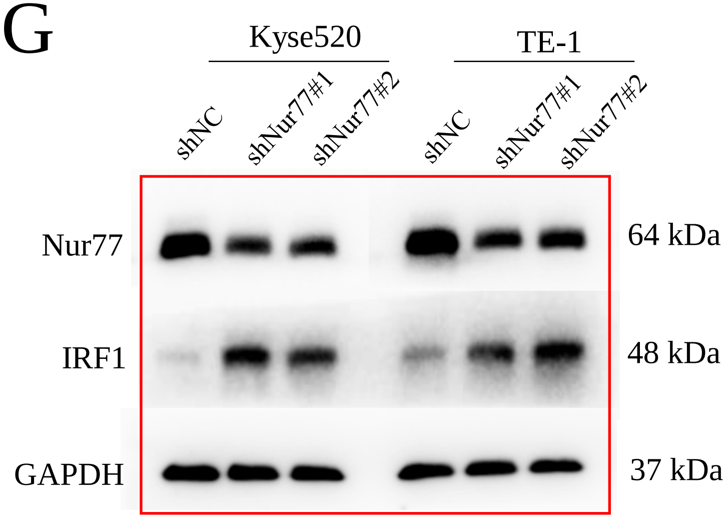
Kyse520
TE-1
shNC
shNur77#1
shNur77#2
shNC
shNur77#1
shNur77#2
Nur77
IRF1
GAPDH
64 kDa
48 kDa
37 kDa

Figure 4

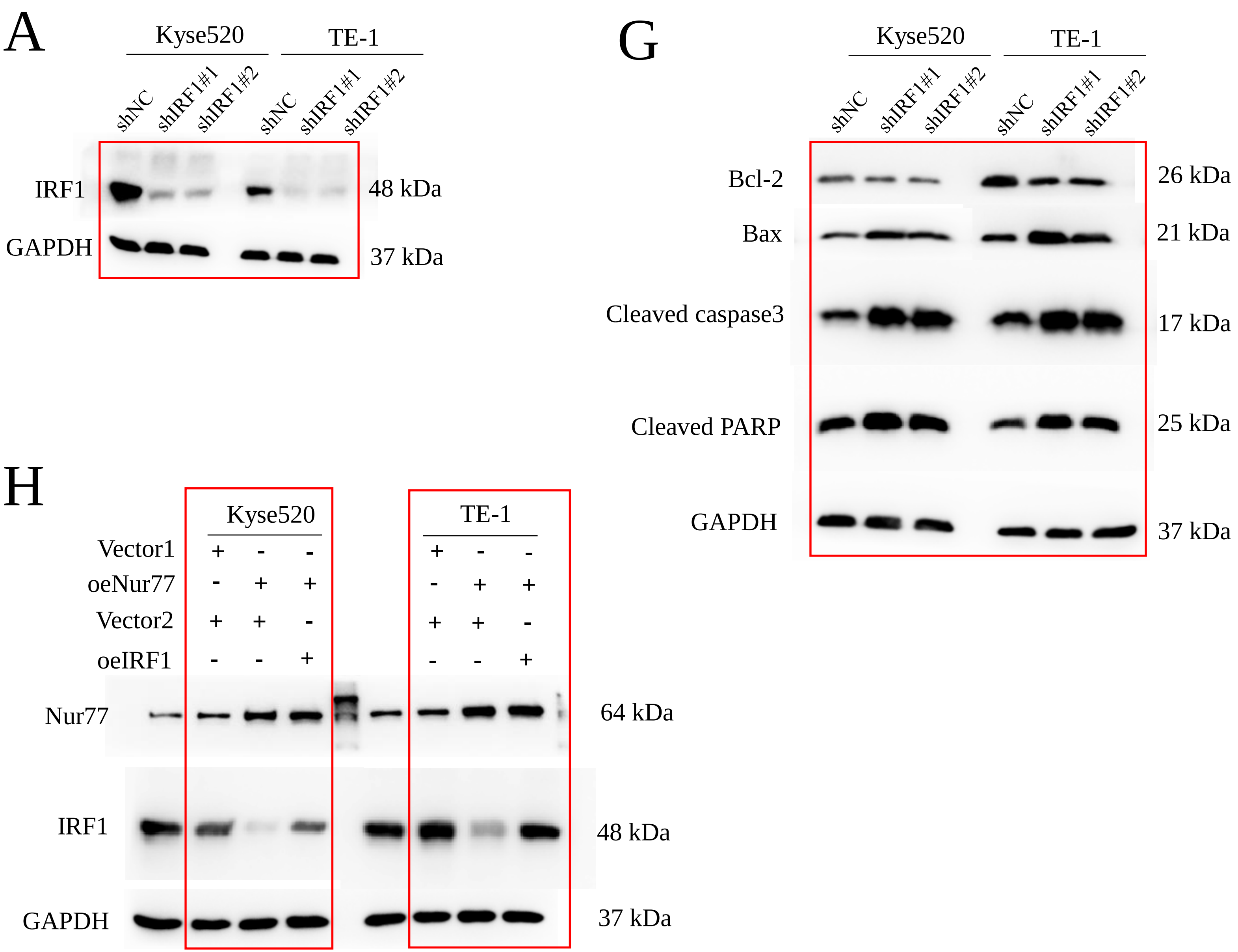
A
Kyse520
TE-1
shNC
shIRF1#1
shIRF1#2
shNC
shIRF1#1
shIRF1#2
IRF1
GAPDH
48 kDa
37 kDa
G
Kyse520
TE-1
shNC
shIRF1#1
shIRF1#2
shNC
shIRF1#1
shIRF1#2
Bcl-2
Bax
Cleaved caspase3
Cleaved PARP
GAPDH
26 kDa
21 kDa
17 kDa
25 kDa
37 kDa
H
Kyse520
TE-1
Vector1 + - - + - -
oeNur77 - + + - + +
Vector2 + + - + + -
oeIRF1 - - + - - +
Nur77
IRF1
GAPDH
64 kDa
48 kDa
37 kDa

Figure 5

D

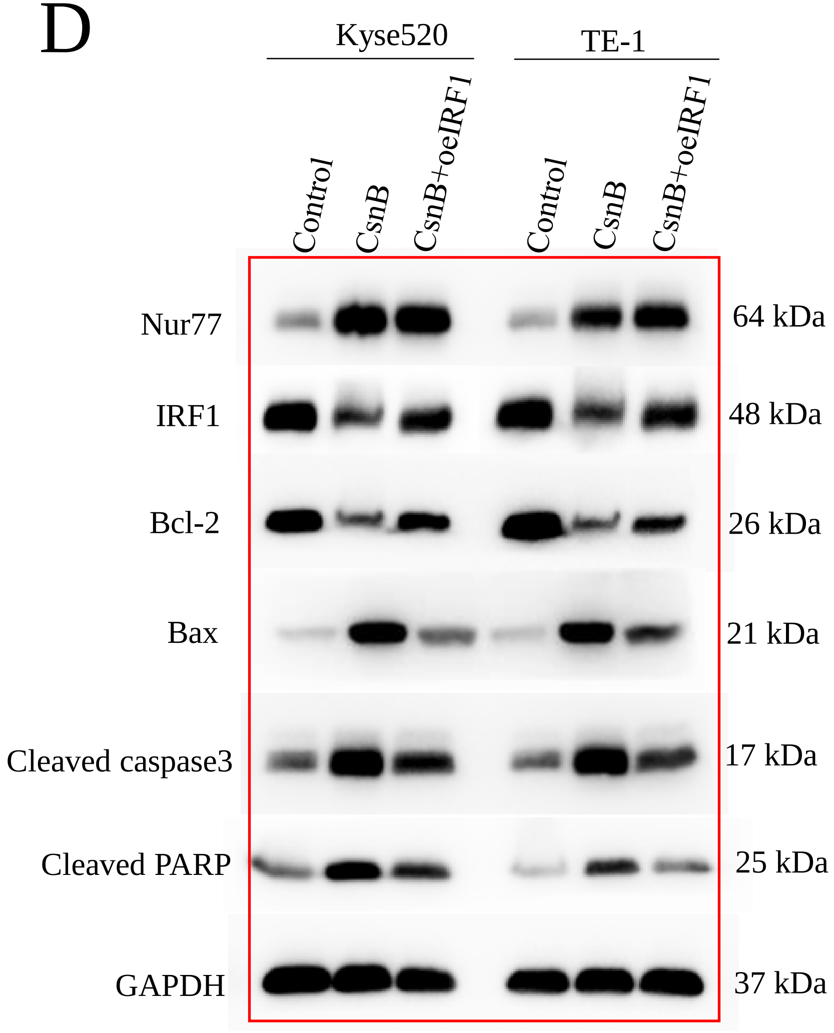
Kyse520
TE-1
Control
CsnB
CsnB+oeIRF1
Control
CsnB
CsnB+oeIRF1
Nur77
IRF1
Bcl-2
Bax
Cleaved caspase3
Cleaved PARP
GAPDH
64 kDa
48 kDa
26 kDa
21 kDa
17 kDa
25 kDa
37 kDa

I

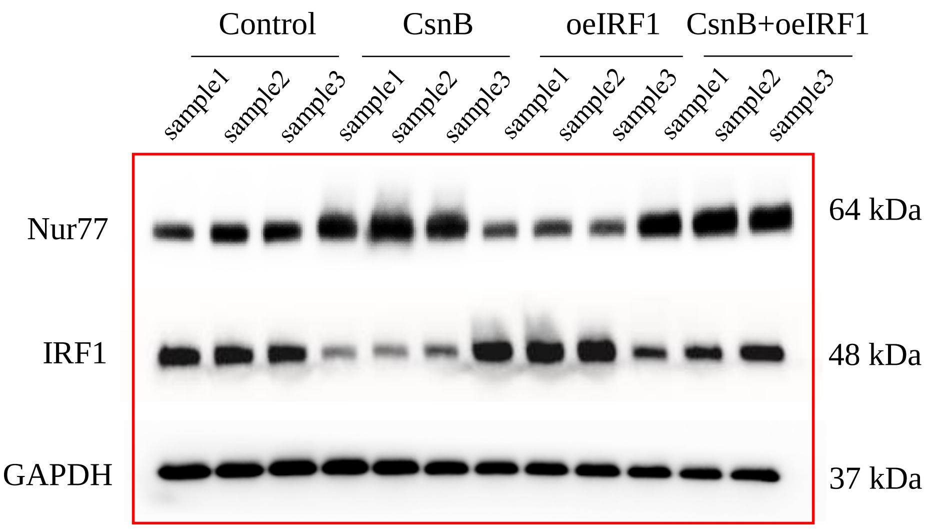
Control
CsnB
oeIRF1
CsnB+oeIRF1
sample1
sample2
sample3
sample1
sample2
sample3
sample1
sample2
sample3
sample1
sample2
sample3
Nur77
IRF1
GAPDH
64 kDa
48 kDa
37 kDa

Figure 6

B

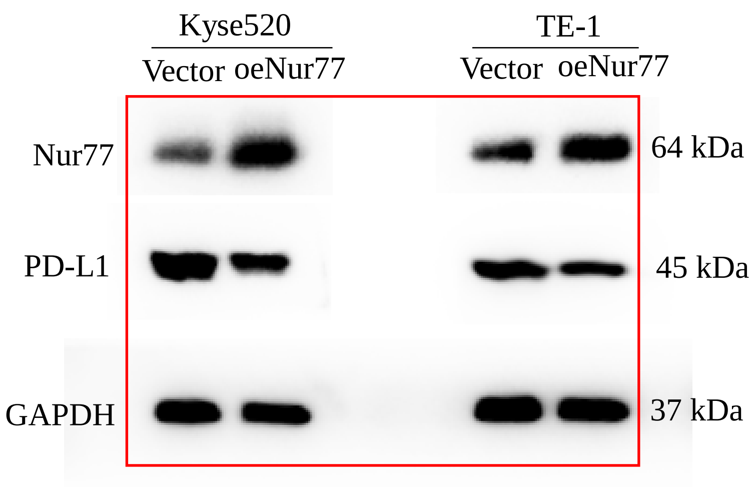

Kyse520
TE-1
Vector
oeNur77
Vector
oeNur77
Nur77
PD-L1
GAPDH
64 kDa
45 kDa
37 kDa

D

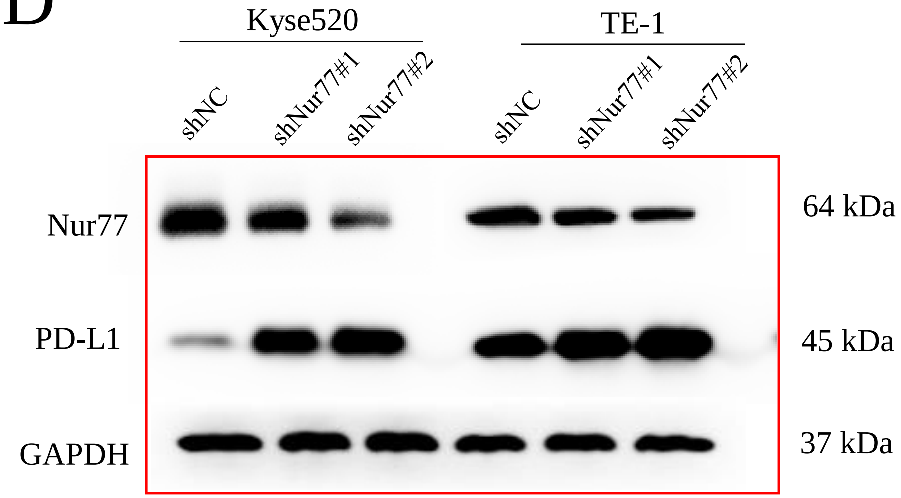

Kyse520
TE-1
shNC
shNur77#1
shNur77#2
shNC
shNur77#1
shNur77#2
Nur77
PD-L1
GAPDH
64 kDa
45 kDa
37 kDa

Figure 7

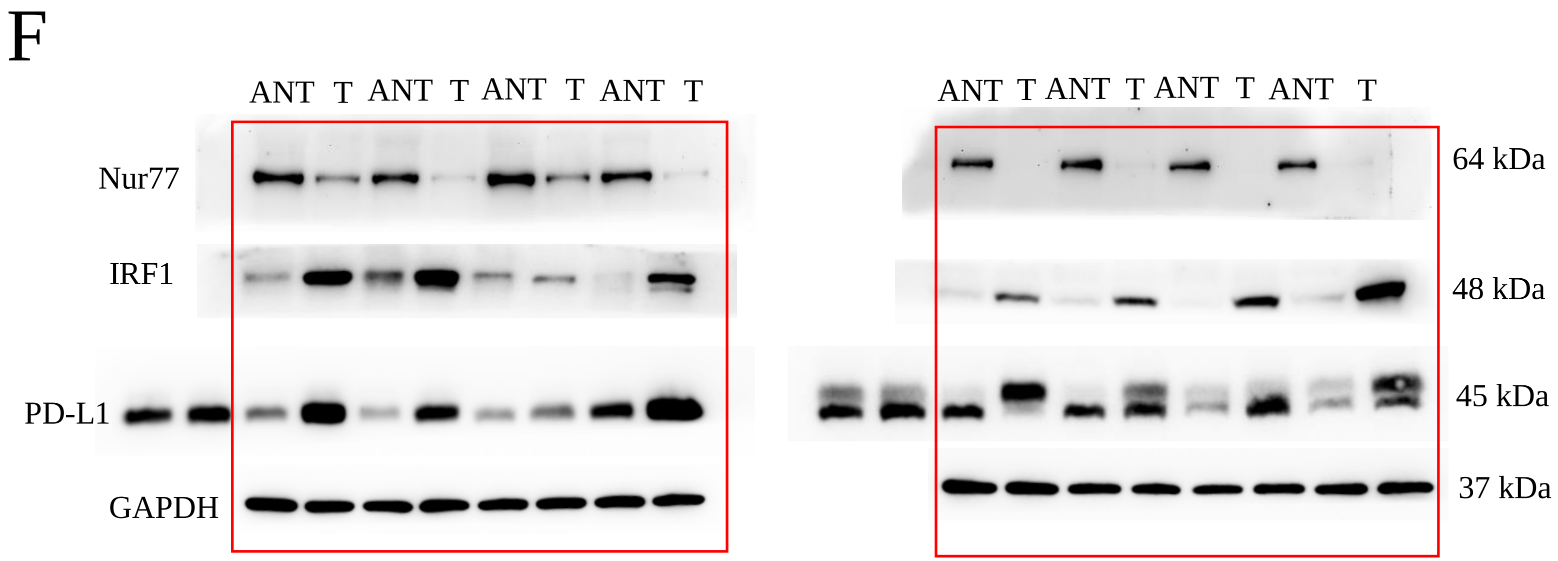
F
ANT T ANT T ANT T ANT T
ANT T ANT T ANT T ANT T
Nur77
IRF1
PD-L1
GAPDH
64 kDa
48 kDa
45 kDa
37 kDa

SFigure 1

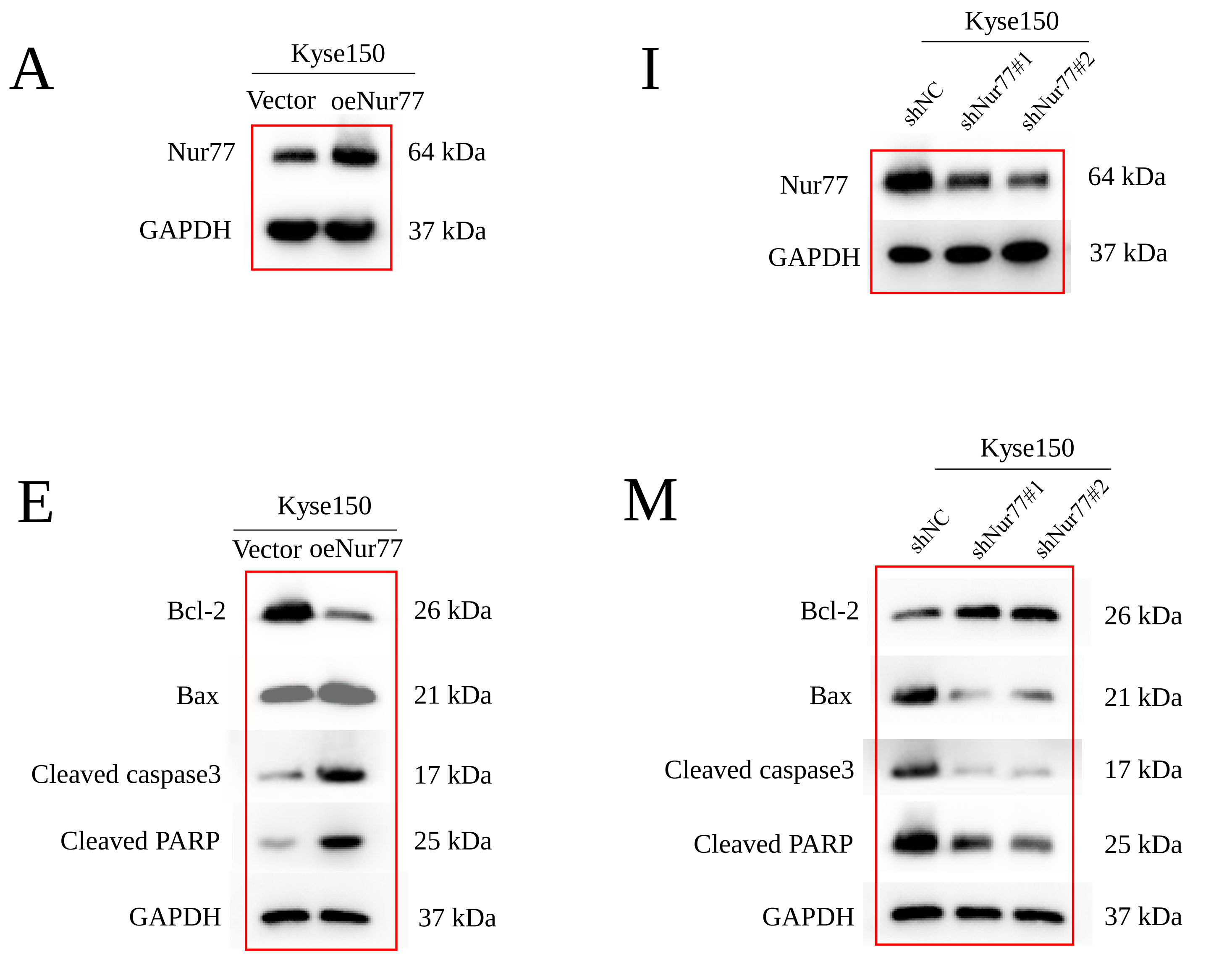
A
Kyse150
Vector
oeNur77
Nur77
64 kDa
GAPDH
37 kDa
I
Kyse150
shNC
shNur77#1
shNur77#2
Nur77
64 kDa
GAPDH
37 kDa
E
Kyse150
Vector oeNur77
Bcl-2
26 kDa
Bax
21 kDa
Cleaved caspase3
17 kDa
Cleaved PARP
25 kDa
GAPDH
37 kDa
M
Kyse150
shNC
shNur77#1
shNur77#2
Bcl-2
26 kDa
Bax
21 kDa
Cleaved caspase3
17 kDa
Cleaved PARP
25 kDa
GAPDH
37 kDa
